# Flexible Textile-Based Hybrid Piezoresistive Sensors for Human-Motion Monitoring

**DOI:** 10.3390/polym18182205

**Published:** 2026-09-10

**Authors:** Hatice Aylin Karahan Toprakci, Mukaddes Sevval Cetin, Ozan Toprakci

**Affiliations:** 1 Department of Polymer Materials Engineering, Yalova University, Yalova 77200, Turkey; ozan.toprakci@yalova.edu.tr; 2 Institute of Graduate Studies, Yalova University, Yalova 77200, Turkey; sevvalcetin1040@gmail.com

**Keywords:** textile-based hybrid piezoresistive sensor, flexible piezoresistive strain sensor, carbon nanofiber, carbon black, poly[styrene-b-(ethylene-co-butylene)-b-styrene] (SEBS)

## Abstract

Textile-based sensors offer a promising platform for human-motion monitoring because of their flexibility, comfort, and ease of integration into clothing. The objective of this study was to develop a textile-based hybrid piezoresistive sensor by combining carbon black (CB) and carbon nanofibers (CNFs) with different filler geometries within a flexible poly[styrene-b-(ethylene-co-butylene)-b-styrene] (SEBS) matrix. The novelty of the proposed approach lies in incorporating CB and CNFs into the same elastomeric sensing layer directly deposited onto an elastic knitted textile substrate and systematically comparing its sensing behavior with the corresponding single-filler CB/SEBS and CNF/SEBS systems. Three sensing coatings, CB/SEBS, CNF/SEBS, and hybrid CB+CNF/SEBS, were deposited onto textile substrates by blade coating. All formulations exhibited uniform coating and good adhesion to the fabric. Coating uniformity and adhesion were assessed through visual inspection, electron microscopy, and mechanical cycling by monitoring peeling, cracking, and delamination. Dynamic piezoresistive tests demonstrated that all sensors were capable of strain monitoring, while the 2 wt% CB+ 2 wt% CNF hybrid formulation exhibited the highest sensitivity among the investigated systems. The resistance response was dependent on both the magnitude and rate of strain deformation. The hybrid sensor was further successfully applied to monitor knee, wrist, and elbow movements during physical exercise. These findings demonstrate that the combination of CB and CNF fillers within a flexible SEBS matrix provides a promising route toward highly strain-sensitive textile sensors for wearable human-motion monitoring.

## 1. Introduction

Textiles are attractive platforms for wearable electronics because of their flexible, lightweight, and comfortable structures. Wearable electronic systems can broadly be considered as rigid devices attached to textiles or as flexible electronic functions integrated within or onto the textile structure. Rigid devices may add bulk, weight, discomfort, and perspiration-related limitations, whereas structurally integrated flexible electronics can better preserve the conformability and comfort expected from clothing [1,2,3]. This group of textile products are called electronic textiles (e-textiles) and the mostly considered functions for these structures can be given as actuating [4], energy harvesting [5,6], and sensing [3]. In these applications, sensing has become quite important recently. Textile-based sensors are e-textiles that integrate various electronic mechanisms and functions to monitor any type of physiological or environmental variable. Although early textile-sensing studies appeared in the late 1990s and early 2000s [7,8,9,10,11], interest has increased substantially with the growth of wearable electronics. Piezoresistive sensing is particularly attractive for strain monitoring because mechanical deformation can be detected through a change in electrical resistance or conductance. Textile-based piezoresistive sensors (TBPSs) have therefore been widely investigated for monitoring strain, bending, force, and body motion [3,12].

There are some requirements for obtaining TBPSs with good performance. First, electrically conductive materials should be incorporated into or onto the textile structure [13] or processed directly into textile forms, such as conductive fibers [14]. Conductive networks can be created using various materials and fabrication techniques, including knitting, weaving, printing, and coating. Among these approaches, printing and coating are particularly attractive because of their relatively low cost, ease of fabrication, flexibility, stability, and sensing performance. Second, the conductive sensing material should respond sensitively to external stimuli such as strain or pressure. Third, the sensing system should maintain a stable performance under repeated deformation, with good recoverability and resilience. For printed or coated TBPSs, strong adhesion between the sensing layer and the textile substrate is also essential and can be improved through the use of polymeric matrices with suitable adhesion properties. Because wearable sensors are expected to become increasingly integrated into daily life for applications ranging from health monitoring to sleep tracking, practical requirements related to everyday clothing should also be considered during sensor design. These include the mechanical limits of the garment, wearer comfort, and the location of the sensor on the textile structure. From this perspective, fabrics with high flexibility and resilience are particularly promising substrates. Elastic fabrics can conform closely to the body while maintaining comfort and adapting to repeated body movements. The flexibility and recoverability of a fabric depend strongly on its construction and fiber composition. Knitted fabrics containing elastomeric fibers generally exhibit high extensibility and good elastic recovery. In such systems, the conductive sensing layer is commonly coated or printed onto the fabric surface. Therefore, the mechanical compatibility between the textile substrate and the conductive sensing layer should also be considered. When the substrate and sensing layer exhibit compatible deformation behavior and strong interfacial adhesion, applied stress can be transferred more uniformly from the fabric to the piezoresistive sensing layer, resulting in a more repeatable and stable electromechanical response [2,15].

The piezoresistive response of conductive polymer-based sensors is influenced by filler morphology, concentration, dispersion, matrix properties, substrate architecture, and fabrication method. Electrical transport occurs through filler–filler contacts and electron tunneling, while deformation alters contact density, inter-filler distance, and filler orientation. These mechanisms vary depending on material architecture and loading conditions. Sam-Daliri et al. reported f-GNP/epoxy sensors governed by direct contact and tunneling between GNPs [16], while Li et al. showed that both bulk and contact resistance contributed to the response of c-MWCNT/TPU pressure sensors [17]. Similarly, Syed et al. demonstrated that strain altered conductive pathways and inter-filler distances in SCF/PP composites [18]. Therefore, piezoresistive performance should be evaluated considering the conductive network, sensor architecture, and loading conditions rather than sensitivity alone. Based on these considerations, the selection of conductive fillers and their integration into textile substrates play a critical role in determining the sensing behavior of TBPSs. Various materials have been employed as sensing layers for the development of TBPSs, including inherently conductive polymers [19,20] and conductive polymer composites [3,21,22,23,24,25,26,27,28,29,30,31,32]. In most of these studies, a single conductive filler was used, such as graphite [25,26], carbon black (CB) [21,24,27], carbon nanofibers (CNFs) [3,22], graphene [23,30,33], multilayer graphene [28], reduced graphene oxide (rGO) [31,32], carbon fibers [18], carbon nanotubes (CNTs) [34,35,36,37], and liquid metals [38,39].

In addition to single filler-containing systems, hybrid fillers can also be used to tune the electrical and mechanical properties of the system. They are of significance in terms of combining advantages of different materials. In this way, electrical, mechanical, and electromechanical performance (sensitivity, stability) are improved with cost-effectiveness and enhanced process performance. However, it is relatively new and there are few studies about textile-based hybrid piezoresistive sensors (TBHPSs). TBHPSs are fabricated by using conductive composites that contain at least two different fillers that can be selected from carbon-based fillers [13,14,40,41], metal-based fillers [41,42], inherently conductive polymers (ICPs) [14,43,44], or nonconductive fillers [45]. To optimize some properties, and increase the sensitivity of the sensor, fillers with various morphologies and aspect ratios can be mixed together or coated/printed on the textile substrate layer by layer [41].

As this study focuses on hybrid fabric sensors, for the literature review, only hybrid fabric sensors fabricated through the incorporation of different types of fillers are discussed. Unlike flexible polymeric sensors, there are a limited number of studies in this area. One of the earliest studies about hybrid fabric sensors focused on the spray-assisted coating of CNTs and rGO hybrid conductive fillers on a polyethylene terephthalate (PET) woven fabric layer by layer. In the second step, zinc oxide (ZnO) nanowire arrays were grown on hybrid fillers with various morphologies. The piezoresistive sensing behavior of the fabric sensors was investigated under various bending strain values ranging from 2.5 to 6.2% as a function of ZnO type, and the gauge factor (GF) was reported to be between 1 and 8. Sensors were used for elbow-movement monitoring under various bending angles (30–120°) and the textile sensor exhibited greater current changes at larger bending angles, enabling the detection and quantification of bending strain [40]. In another study, cotton (Co) fabric was coated with graphene oxide and chemical reduction was carried out for rGO formation. Afterward, the fabric was dip-coated with varying amounts of single-walled carbon nanotubes (SWCNTs), and the piezoresistive response of the samples was investigated under bending strains ranging from 4.4% to 11.6%. GF values between 2.5 and 6 were obtained, and a negative piezoresistive response was observed. Sensor gloves were found successful in terms of monitoring wrist movement, finger pressing, bending, and grabbing [13]. Another interesting study focused on the development of textile sensors based on PET woven elastic bands (PEBs). The band was dip-coated with SWCNTs, and after that, the sample was coated with a silver (Ag) paste. The Ag/SWCNT/PEB sensor fabric showed positive piezoresistance, high repeatability, and GF around 3550 in the strain range of 1.5–5% due to the high resilience of the fabric sensor. Additionally, hybrid sensors were used for firefighting gloves and found successful for the cyclic motion detection of fingers [41]. Altaf et al. prepared textile sensors by layer-by-layer spray coating of silver nanoparticles (AgNPs) and SWCNTs onto PET/Co fabrics. Three different sensor configurations were prepared using fabrics with and without pre-strain. The most sensitive sensor exhibited a positive piezoresistive response, with a GF of approximately 27000 at 50% strain [42]. Souri and Bhattacharyya prepared TBPSs using Co and wool fabrics as substrates for a hybrid sensing layer composed of graphene nanoplatelets (GNPs) and CB. The fabrics were immersed in an ultrasonication bath containing the GNP/CB hybrid fillers, which facilitated the deposition and adhesion of the conductive fillers onto the fabric surface. Subsequently, conductive fabrics were coated with a silicone elastomer layer. TBPSs were tested under various deformation conditions and exhibited a positive piezoresistive response. The GF ranged from approximately 1.64 to 20.4 for Co-based sensors and from 0.4 to 4.26 for wool-based sensors at strains of up to 75%, while maintaining good stability. The sensors were also successfully used to detect different human motions, including wrist and finger flexion [46].

As discussed above, carbon/carbon and carbon/metal combinations were predominantly employed as hybrid fillers. However, in some studies, ICPs, such as poly(3,4-ethylenedioxythiophene):polystyrene sulfonate (PEDOT:PSS), polyaniline (PANI), and polypyrrole (PPy), have also been incorporated into hybrid structures. For example, CNT/PEDOT:PSS/natural rubber fibers were fabricated, sewn onto fabric, and used as TBPSs. The sensor exhibited high stretchability and a highly linear piezoresistive response even at strains of up to 1000%. Both CNT content and strain ratio were reported to significantly influence sensor sensitivity, with GF values ranging from 3.6 to 4 [14]. In another study, PANI was polymerized onto Co/spandex fabric, followed by the deposition of Ag via an electroless plating process. The resulting TBPSs showed a positive piezoresistive response. The PANI-coated sensor exhibited a GF of approximately 6.4, whereas the PANI/Ag sensor showed a considerably higher GF of approximately 26 at 2.4% strain, together with good resilience and stability. Additionally, the PANI/Ag-based TBPS demonstrated good antimicrobial activity [47]. In a separate study, PPy was used as the ICP. First, polydopamine (PDA) was polymerized onto Co fabrics. Subsequently, PDA-coated Co fabrics were immersed in SWCNT dispersion under ultrasonication to facilitate the deposition of SWCNTs onto the fabric surface. After drying, SWCNT/PDA/Co fabrics were incorporated into pyrrole suspension for the polymerization of PPy. Finally, PPy/SWCNT/PDA/Co TBHPSs were fabricated using layer-by-layer coating. The resulting sensor showed a positive piezoresistive response, with a GF of approximately 9.5 at 5% strain. In addition, the hybrid textile sensors were successfully employed for monitoring various human motions [43]. Peng et al. developed hybrid piezoresistive textile sensors using a nylon strip (NS) as the substrate. The NS was coated with AgNPs and PPy, while polydimethylsiloxane (PDMS) was applied as a protective layer. The resulting TBHPS showed a positive piezoresistive response and remained sensitive over a strain range of up to 70%, with a GF of 1.61 × 10^3^. The sensor also demonstrated good durability, maintaining a stable performance after 5000 loading–unloading cycles. Owing to the hydrophobic PDMS coating, TBHPS was successfully used to detect various joint motions even under harsh environmental conditions [48].

In addition to conductive phases, nonconductive components can also be incorporated into the sensing layer. In one such study, thermoplastic polyurethane (TPU) nonwoven fabric was used as the textile substrate. TBHPS was prepared by dip-coating the fabric with a cellulose nanocrystal (CNC)/graphene mixture. The resulting sensors exhibited a high GF up to 2.36 × 10^4^ at 98% strain [49]. Another hybrid textile sensor incorporating conductive and nonconductive fillers was developed by Zhu et al. using electrospun TPU membranes as the substrate. Hybrid CNC/CNT dispersions with different composition ratios were pumped and filtered onto the TPU nonwoven membranes to form the sensing layer. The resulting sensors exhibited a positive piezoresistive response, with the highest GF reported to be 321 at a strain level of 500%. The highly stretchable TPU-based hybrid textile sensors were also successfully employed for monitoring various body motions, ranging from respiration to knee bending [45]. A recent research focus on textile-based sensors is the development of multifunctional systems with fire-protection capabilities for early fire warning and physiological monitoring during firefighting, with the aim of enhancing wearer safety. To impart flame-retardant (FR) properties, Guo et al. coated PET fabrics with MXene and phosphorus-containing flame retardants. Although a second conductive filler was not incorporated into the MXene sensing layer, the FR components acted as additional functional phases within the hybrid structure. The MXene-based layer exhibited not only a positive piezoresistive response but also thermoelectric behavior. As a result, a flame-retardant piezoresistive textile sensor with fire-warning and thermoelectric ability was developed [50].

In this study, the design and fabrication of a TBHPS were performed with an elastic fabric and a hybrid nanocomposite sensing layer. Vapor-grown carbon nanofibers (CNFs) and carbon black were used as the conductive hybrid fillers and poly[styrene-b-(ethylene-co-butylene)-b-styrene] (SEBS) was used as the flexible matrix. Morphological, mechanical, and electromechanical properties were analyzed. Additionally, TBHPS was used to monitor various human body motions, such as knee, wrist, and elbow movements, during physical exercise. To the extent of the present literature, no study was found in the literature about CB+CNF/SEBS-based hybrid piezoresistive textile sensors. The abovementioned studies demonstrate that the performance of hybrid textile piezoresistive sensors cannot be evaluated solely based on GF because sensitivity is strongly coupled to sensor architecture, fabrication route, substrate, and working strain range. For example, multilayer metal/carbon systems such as Ag/SWCNT-based sensors can exhibit very high GF values within relatively narrow strain ranges [41], whereas highly stretchable hybrid architectures based on elastomeric or fibrous substrates can operate at much larger strains but with lower GF values [14]. Similarly, GNP/CB-coated textile sensors provide a carbon/carbon hybrid architecture [46] but differ from the present system in both the conductive-filler geometry and fabrication strategy. Therefore, a meaningful comparison of hybrid sensors should consider not only GF, but also working strain range, conductive-network architecture, cyclic recoverability, fabrication complexity, and compatibility with the textile substrate.

As shown above, previously reported hybrid textile sensors require relatively complex fabrication procedures, such as layer-by-layer spray coating, multiple dip-coating steps, ultrasonication, electroless metal plating, or in situ polymerization of ICPs, which may increase processing time and complexity and can make it more difficult to achieve uniform and reproducible coatings over textile surfaces. In addition, multilayer or weakly adhered sensing layers may be susceptible to surface wear, material shedding, cracking, or delamination during repeated bending and stretching, potentially affecting long-term sensing stability. In contrast, the blade-coating approach used in the present study provides a comparatively simple route for depositing the SEBS-based sensing layer onto the textile substrate. Electron microscopy, visual examination, and mechanical cycling indicated good coating continuity and adhesion without apparent peeling, cracking, or delamination under the investigated conditions. The relatively simple coating procedure may also offer advantages for future scale-up compared with multi-step fabrication routes. The novelty of this study lies in the use of a CB+CNF/SEBS hybrid sensing system that, to the best of our knowledge, has not previously been reported for textile-based piezoresistive sensors. CB and CNFs were selected as hybrid conductive fillers because of their markedly different geometries and aspect ratios. CB consists of approximately spherical particles with an aspect ratio close to 1, whereas CNFs possess a fibrous morphology with aspect ratios exceeding 100. Their combination is therefore expected to provide complementary contributions to the formation and deformation of the conductive network. Although high-aspect-ratio fillers can facilitate the formation of conductive pathways at relatively low filler concentrations, their large surface area and rigid fibrous structure may substantially increase the viscosity of the coating formulation and hinder homogeneous dispersion. This may adversely affect filler distribution, coating uniformity, and sensing reproducibility. Therefore, CB was combined with CNFs to reduce the amount of high-aspect-ratio filler required while promoting more homogeneous dispersion through high-shear planetary mixing and maintaining an effective conductive network. This hybrid filler design was intended to improve both processability and piezoresistive sensing performance.

This architecture produces a heterogeneous conductive network containing CB–CB, CNF–CNF, and CB–CNF junctions within a single deformable sensing layer. Under tensile deformation, changes in filler contacts and inter-filler separation, together with the translation and rearrangement of CB particles and rotation, slippage, alignment, and reorientation of CNFs, are expected to contribute to piezoresistance. Experimentally, the hybrid sensor exhibited a GF of approximately 108 at 20% strain and maintained positive piezoresistive sensing up to approximately 25% strain at 5 mm/min. Thus, the present design targets a combination of useful sensitivity, deformation range, cyclic stability, and simple, affordable coating-based fabrication for wearable joint-motion monitoring rather than the maximization of GF alone. Additionally, improved weather resistance of SEBSs further contributes to the novelty of the present study.

## 2. Materials and Methods

SEBS (Kraton G1643), with an S/EB ratio of 19/81 and a melt flow index of 75 g/10 min (5 kg load at 200 °C), was used as the polymer matrix. CB (particle size: 30–50 nm; aspect ratio: ~1; Timcal Super C65, MTI) and CNFs (fiber diameter: 125–150 nm; fiber length: 20–200 µm; aspect ratio: 130–1600; Sigma-Aldrich, St. Louis, MO, USA) were used as conductive fillers. Toluene (Merck) was used as the solvent. The fabric used for the textile sensors was obtained from commercially available compression socks with a high spandex content. The knitted elastic fabric consisted of 25/75 spandex/polyamide and had an areal density of 292 g/m^2^.

In the first step, SEBS was dissolved in toluene at an SEBS/toluene weight ratio of 1/4 for 12 h at ambient temperature using a magnetic stirrer (MSH-20D, DAIHAN Scientific Co., Ltd., Wonju, Republic of Korea) at 700 rpm. CB, CNF, and hybrid CB+CNF fillers with a CB/CNF weight ratio of 1/1 were premixed with 5 g of toluene using a high-shear planetary mixer (Kurabo Mazerustar KK 250, Kurabo Industries Ltd., Osaka, Japan) for 90 s at 1500 rpm and subsequently mixed with the polymer solution under the same conditions. Three sensor formulations were prepared using conductive SEBS composites containing 4 wt% CB, 4 wt% CNF, and 2 wt% CB + 2 wt% CNF. Three sensor formulations were prepared using conductive SEBS composites containing 4 wt% CB, 4 wt% CNF, and 2 wt% CB + 2 wt% CNF. The total filler concentration was selected based on our previous study, in which the suitable sensing region for CB/SEBS and CB+CNF/SEBS composites was determined to be in the range of approximately 4–6 wt% [51], together with preliminary experiments on fabric-based sensors. A total filler concentration of 4 wt% was selected for all formulations to enable a direct comparison among the CB-, CNF-, and hybrid-filled sensors, while maintaining the hybrid composition at a CB/CNF weight ratio of 1:1.

**Figure 1:**
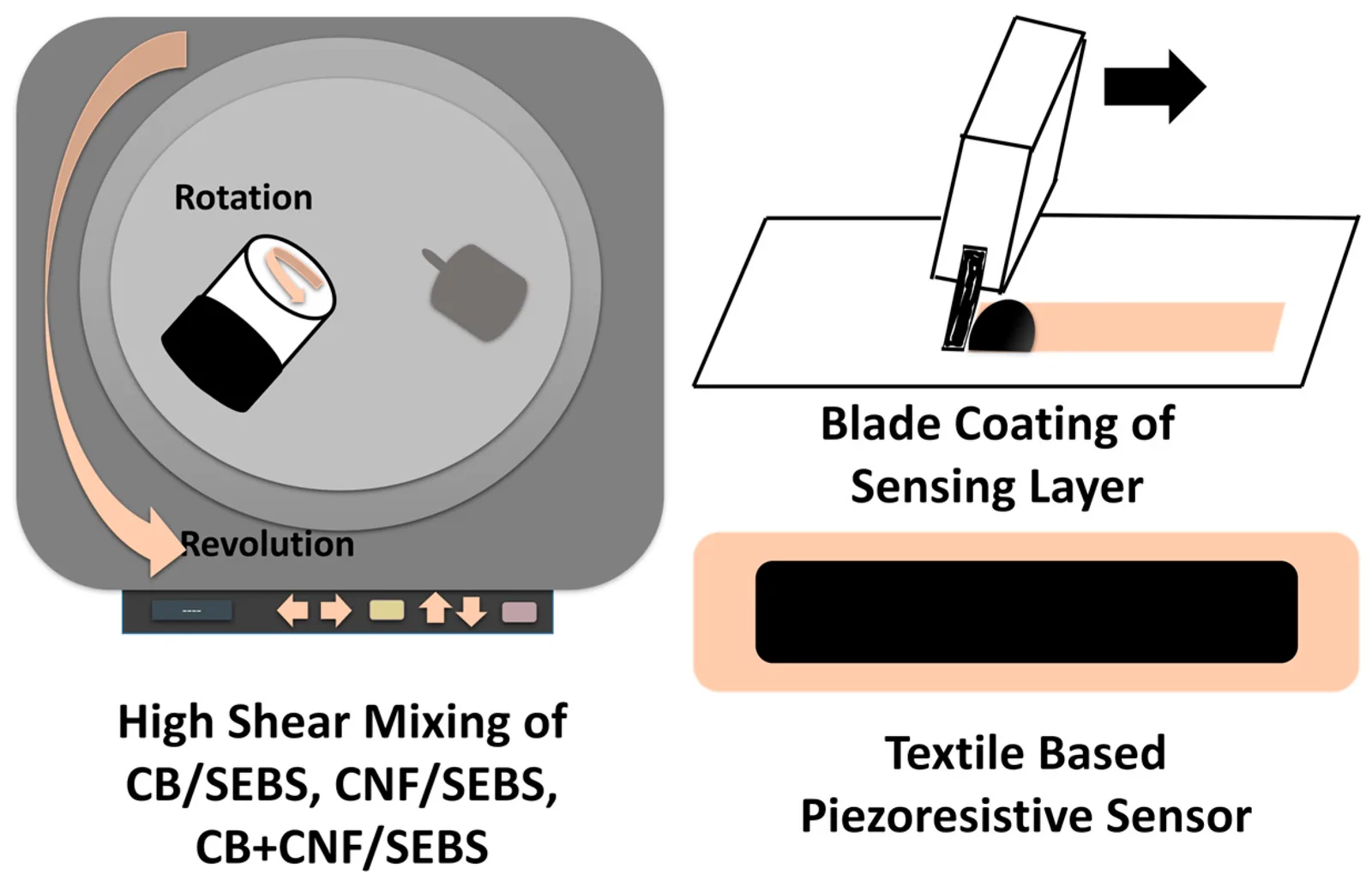
Preparation steps for textile-based sensors.

For TBHPS fabrication, the conductive pastes (CB/SEBS, CNF/SEBS, and CB+CNF/SEBS) were coated onto knitted fabric samples with dimensions of 80 mm × 25 mm. The coated samples were left under ambient conditions for 12 h and subsequently dried in a vacuum oven (Wisd WOV-20, DAIHAN Scientific Co., Ltd., Wonju, Republic of Korea) at 40 °C for 24 h to remove residual solvent. The fabrication process is illustrated in Figure 1. Fabric thickness was measured using a thickness gauge (Asimeto, Taichung City, Taiwan). For morphological characterization, the samples were cut using a blade, and the cross-sections of the sensors were sputter-coated with Au/Pd and examined using a field-emission scanning electron microscope (FESEM; JSM-6400, JEOL Ltd., Tokyo, Japan) at an accelerating voltage of 20 kV.

Electromechanical properties were determined by the 4-probe method. To obtain dynamic resistance data, four Cu leads were attached to the fabric sensor by a conductive glue and dried at room temperature for 24 h. Subsequently, a textile sensor was mounted between the grips of the load frame (DVT, Devotrans, Istanbul, Turkey) with a gauge length of 50 mm. The Cu leads were connected to a current source (Model 6221, Keithley Instruments, Inc., Cleveland, OH, USA) and a nanovoltmeter (Model 2182A, Keithley Instruments, Inc., Cleveland, OH, USA); measurements were performed at an applied current of 1 μA.

All three sensor formulations were initially tested at 20% strain and 5 mm/min under identical conditions to provide a standardized comparison of their electromechanical responses. The 20% strain level was selected as an intermediate deformation level for comparative testing rather than as the maximum extensibility of the textile substrate. The hybrid sensor was subsequently evaluated over a broader strain range (5, 10, 20, 25, and 30%) to determine its strain-dependent sensing behavior and practical positive-piezoresistive operating range. In addition, stepwise piezoresistive measurements were performed on the hybrid sensor at crosshead speeds of 5 and 50 mm/min for five cycles at strain levels of 5, 10, 20, and 25%. Using the resistance data, R/R_0_ and GF values were calculated for each test condition. One sensor specimen from each formulation was subjected to electromechanical characterization. Electrical hysteresis was quantified using an area-based approach adapted from Chen et al. [38,39]. Because the mechanical strain and electrical resistance signals were recorded using independent measurement systems, the two time series were synchronized based on the onset of loading. A constant time offset was applied for each experiment to align the beginning of mechanical deformation with the corresponding electrical response, without scaling the time axis. The normalized resistance change was calculated as

(1) $$\frac{\Delta R}{R_{0}}=\frac{R-R_{0}}{R_{0}},$$

where R_0_ is the initial electrical resistance measured before cyclic deformation. The areas under the loading and unloading branches of the normalized resistance–strain response, A_loading_ and A_unloading_, respectively, were calculated by numerical integration over their common strain range using the trapezoidal rule. The hysteresis magnitude (DH%) was determined as:

(2) $$DH\left(\%\right)=\frac{|A_{loading}-A_{unloading}|}{A_{loading}}\times 100,$$

The absolute area difference was used to express the magnitude of hysteresis irrespective of the direction of the loading–unloading loop. Hysteresis was evaluated for the 1st, 5th, 10th, 15th, and 20th loading–unloading cycles. The 10th cycle was selected as a representative conditioned cycle for comparison among strain amplitudes and deformation rates, while the cycle-dependent hysteresis values are provided in the Supporting Information.

At the end of this study, human-motion measurements involving knee, wrist, and elbow movements during physical exercise were performed using the TBHPS. The electromechanical measurements in the present study were conducted under laboratory ambient conditions, and the effects of temperature and relative humidity were not independently evaluated. Mechanical cycling data obtained during piezoresistance measurements was used for the determination of the unrecovered strain, stress softening, and change in force.

## 3. Results

### 3.1. Morphology

**Figure 2:**
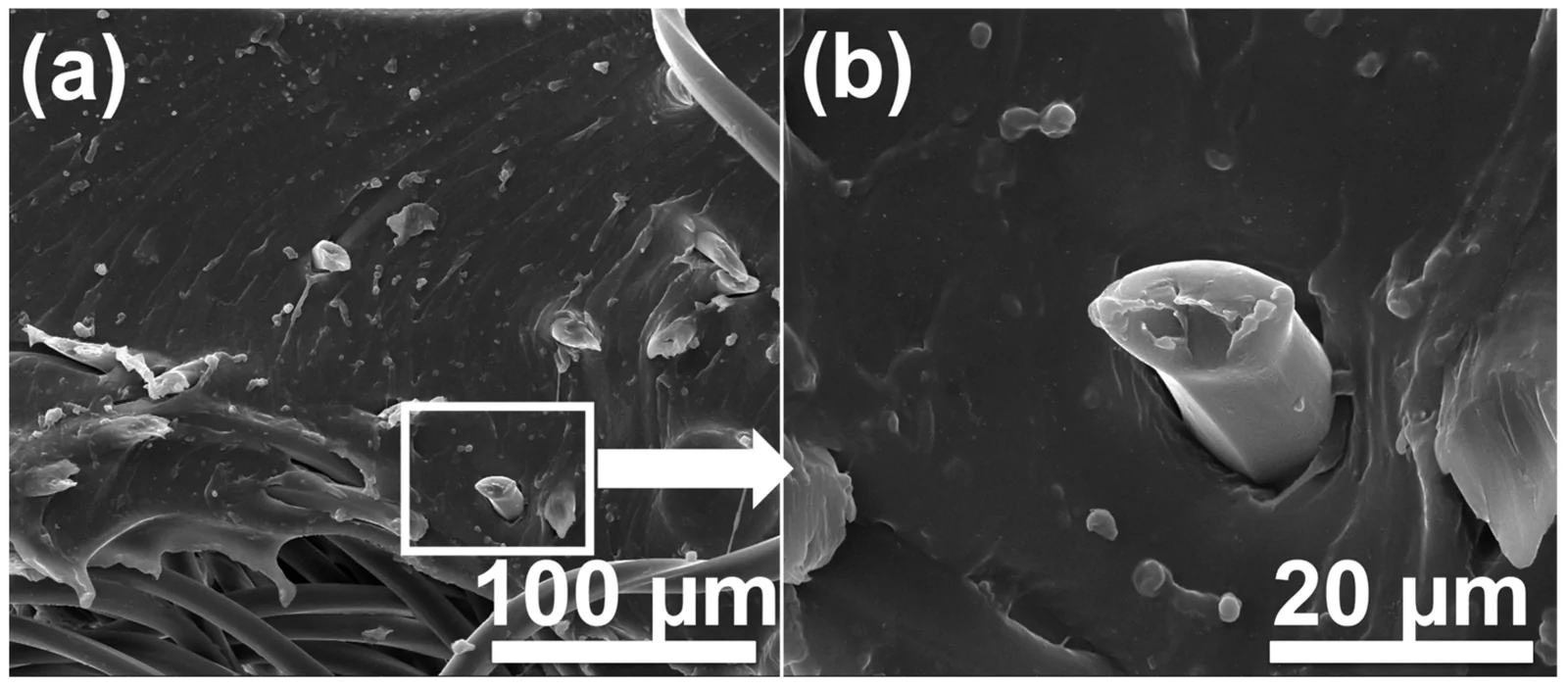
Cross-sectional FESEM images of the CB-based textile piezoresistive sensor at (**a**) 1000× and (**b**) 5000× magnifications.

**Figure 3:**
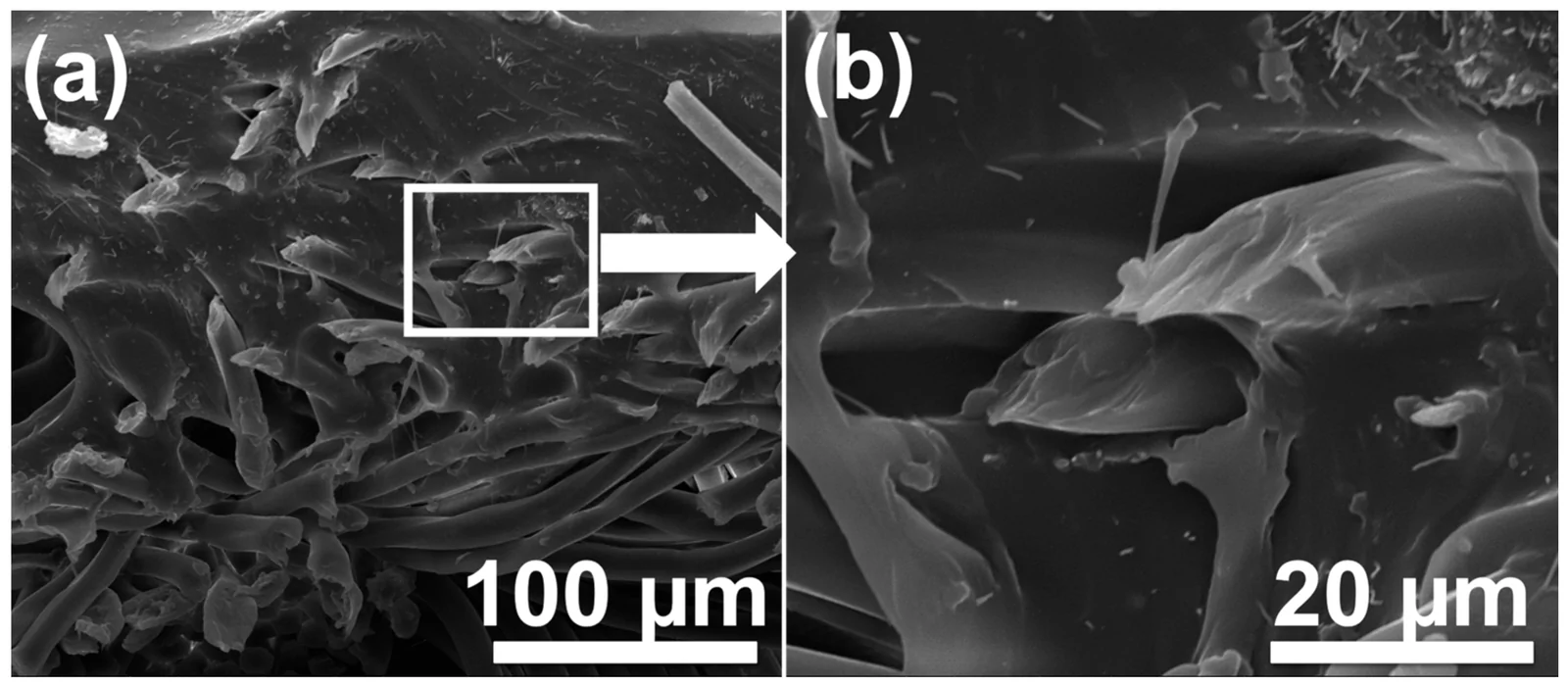
Cross-sectional FESEM images of the CNF-based textile sensor at (**a**) 1000× and (**b**) 5000× magnifications.

**Figure 4:**
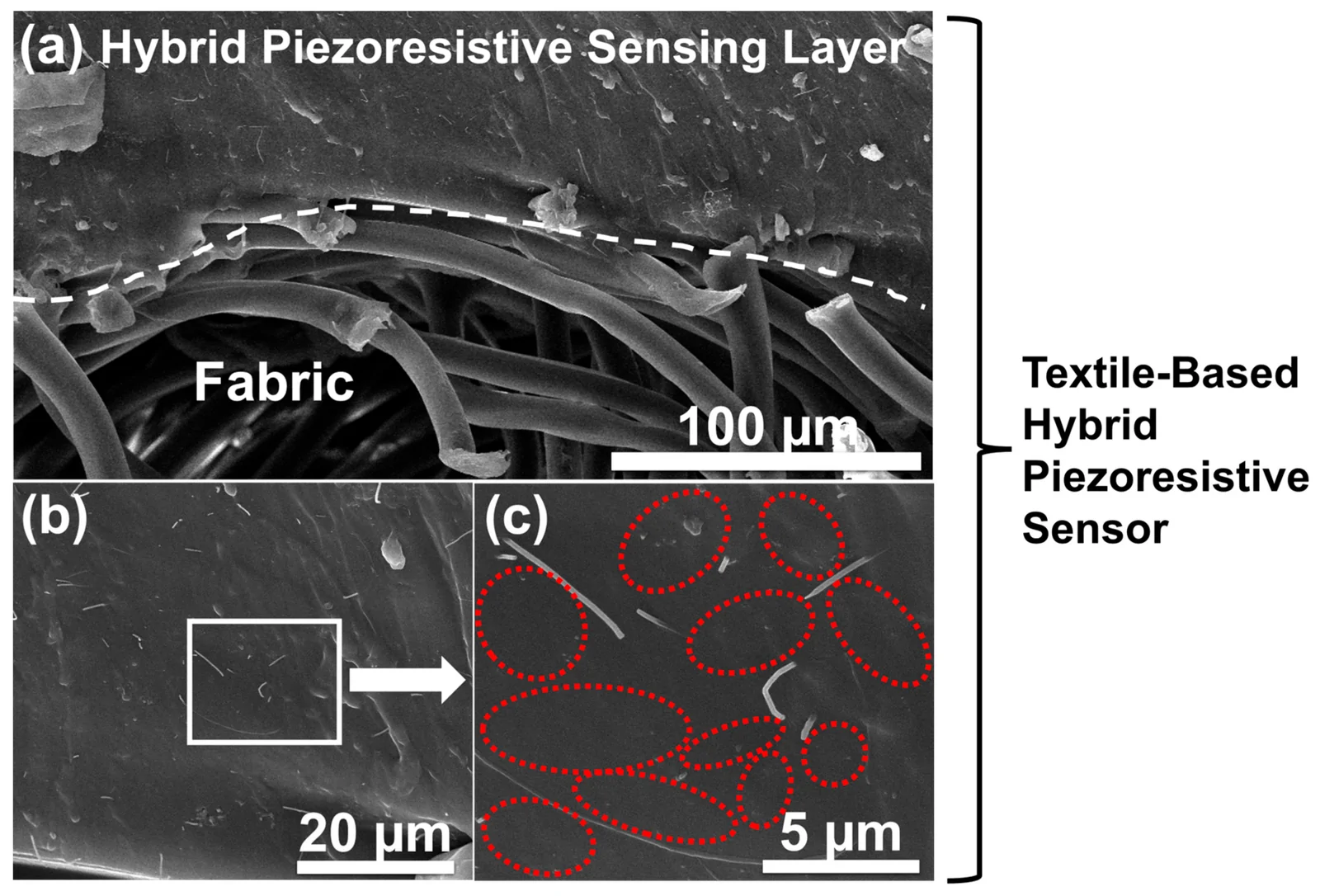
Cross-sectional FESEM images of the TBHPS at (**a**) 1000× (**b**) 5000×, and (**c**) 20000× magnifications. Dotted-line cicrcles: CB.

The microstructural morphology of textile sensors was analyzed by FESEM. In all figures (Figure 2, Figure 3 and Figure 4, Figures S1–S3), there are two basic parts given as the fabric and sensing layer. While fabric consists of continuous fibers the sensing layer consists of a polymeric matrix and fillers. The dark-gray, continuous phase in the sensing layer is the polymeric matrix, and the whitish-gray phases represent the CB or CNF. The cross-sections of the fabrics coated by CB, CNF, and CB+CNF-filled SEBS were examined in terms of fabric-sensing layer interaction as well as piezoresistive layer properties, including filler dispersion, orientation, and filler–matrix interaction. There are some important micro-morphological properties that should be considered for wearable electronics and textile-based sensors fabricated by any type of coating, printing, or deposition process. The properties of the coating layer and layer–fabric interface should be optimum and homogeneous and for a stable system with a meaningful electrical response [3].

In Figure 2a, Figure 3a and Figure 4a, a piezoresistive layer and fabric interaction can be observed. As is obvious from the images, SEBS was the continuous phase and functioned as the binder not only for fillers, but also between the fabric and piezoresistive layer. It was smoothly coated on the fabric surface with a good interface.

It can also be seen that the surface fibers of the knitted fabric are embedded in the sensing layer and are nicely covered, which is very critical in terms of the mechanical properties of the system, especially for sensor stability and durability. If the interface between the two layers is not sufficiently strong, delamination may occur under repeated strain, which can hinder mechanical stress transfer from the fabric to the sensing layer, potentially leading to inaccurate electrical signals. All sensors showed good interfaces between the sensing layer and fabric. Other important points were the filler dispersion, orientation, and filler–matrix interface. Figure 2 shows CB/SEBS-coated fabric sensors. The sensing layer was smoothly coated on the fabric with good adhesion. CB was found to be well-dispersed in the SEBS matrix without any dominant orientation due to its spherical geometry with an aspect ratio of 1. However, some agglomerations of CB clusters were also observed in the percolating network. Figure 3 shows CNF/SEBS-coated fabric sensors. CNFs were well-dispersed throughout the sensing layer without any dominant fiber orientation with good matrix wetting due to the inherited surface roughness of CNFs. A hybrid sensor can be seen in Figure 4. CB and CNF can easily be observed as light-gray parts because of their high electrical conductivity in the dark-gray SEBS phase. CB is a spherical particle, and orientation behavior could be investigated only for CNF. Since the piezoresistive layer was prepared by a high shear mixer based on a solution-based process, CB particles and CNFs were well-dispersed without any dominant orientation. A good filler–matrix interface can be observed more obviously in Figure 4c; both fillers were wet by a SEBS matrix. In addition to this, conductive network formation is significant in terms of piezoresistive response. As can be seen in Figure 4c (circles), CBs fill the gaps between CNFs and CB-CB, CNF-CNF, and hybrid CB-CNF junctions can be observed in the nanocomposite sensing layer.

### 3.2. Electromechanical Properties

**Figure 5:**
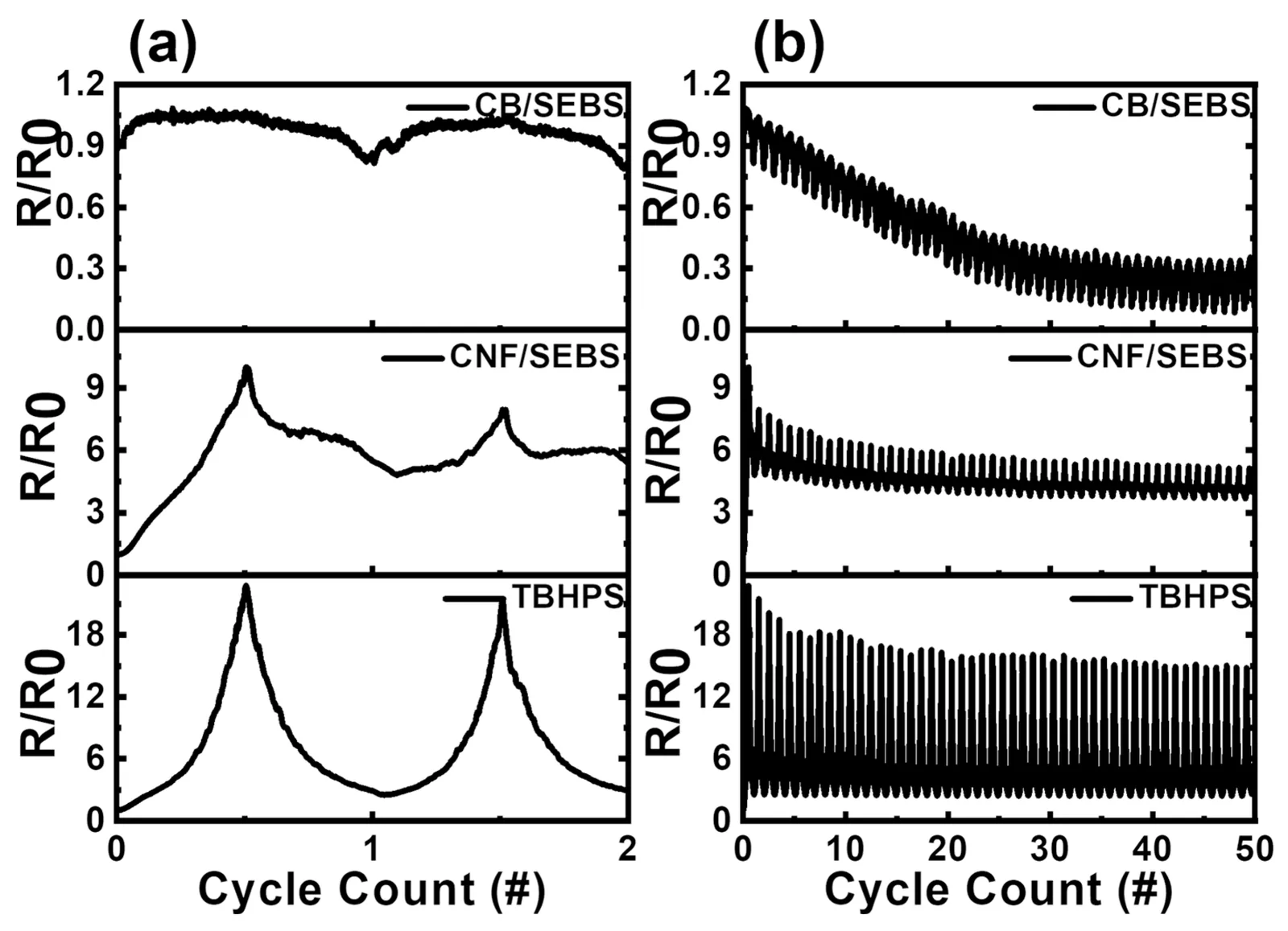
R/R_0_ vs. cycle number for textile sensors under 20% strain at 5 mm/min: (**a**) 2 cycles; (**b**) 50 cycles.

Electromechanical characterization was performed in two steps. In the first step, all samples were cycled at 5 mm/min under 20% strain to observe and compare their performance. After that, TBHPS was analyzed under various conditions and its usability as a wearable sensor for monitoring human movement was investigated. SEBS is one of the most stable thermoplastic elastomers in terms of sensing performance, exhibiting exceptional properties such as flexibility, high resilience, and resistance to ultraviolet degradation, owing to its triblock copolymer structure comprising rigid styrene (S) blocks and soft ethylene–butylene (EB) blocks. Under strain, the S phase functions as the physical crosslinking point, while the EB phase facilitates the material’s stretching, contributing to its superior mechanical properties and durability. During strain loading, the fabric and elastomeric SEBS matrix deform gradually, fibers of the knitted fabric align on the strain axis, and stress is transferred from the fabric to the SEBS matrix and from the SEBS matrix to rigid, conductive fillers. In this case, basically two processes take place: the breakdown of the conductive network and formation of the new conductive network. If the disruption of the network is dominant during stretching, filler–filler separation leads to an increase in resistance, and positive piezoresistance (PP) is observed. On the other hand, if the formation of the new network is dominant, the resistance drops and negative piezoresistance (NP) is observed. In both cases, in addition to filler separation, synergistic effects of the spatial rearrangement of the fillers, such as rotation, slippage, translation, uncurling, alignment, and reorientation, should be considered [3,51]. The piezoresistance for all textile sensors is shown in Figure 5a, as R/R_0_, where R is the resistance at any given time or strain level; R_0_ is the initial resistance value obtained before strain cycling. Figure 5a shows R/R_0_ as a function of cycle number for textile sensors under 20% strain, 5 mm/min for 2 cycles, and all sensos showed PP regardless of filler type, which was an indication of dominant filler separation and an increase in resistance during strain loading. The R/R_0_ values were in the ranges of 0.1–1, 4–9, and 16–20 under 20% strain for CB-based sensor, CNF-based sensor, and TBHPS, respectively. As can be seen from the outcomes, TBHPS shows the best performance in terms of piezoresistance. To observe the cyclic stability of the sensors, 50 cycles were performed, as shown in Figure 5b. All sensors showed good stability, but TBHPS showed the best cyclic stability (TBHPS > CNF-based textile sensor > CB-based textile sensor).

**Figure 6:**
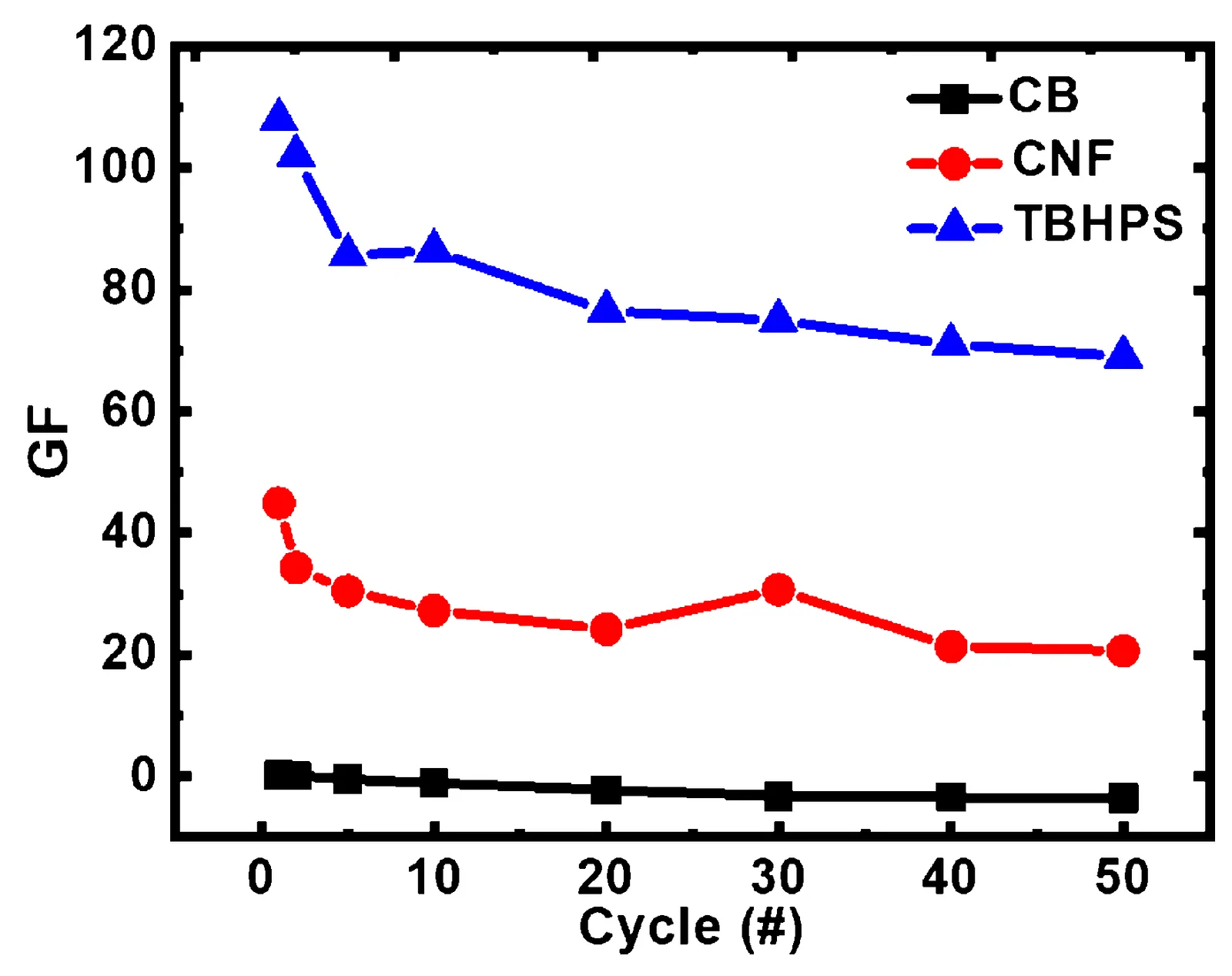
GF values of sensors as a function of cycle number under 20% strain at 5 mm/min.

To observe the sensitivity of the piezoresistive sensors, dynamic GF graphs are presented in Figure 6. GF can be calculated at any strain level through the help of the following formula, where l is the length and l_0_ is the initial length of the sensor.

(3) $$GF=\frac{\Delta R/R_{0}}{\varepsilon }=\frac{\left(R-R_{0}\right)/R_{0}}{\left(l-l_{0}\right)/l_{0}}$$

As shown in Figure 6, the GF values determined from the first loading cycles are approximately 0.2, 45, and 108 for the CB-based sensor, CNF-based sensor, and TBHPS, respectively. The relatively small resistance variation in the CB-based sensor suggests that its conductive CB network remained comparatively stable during deformation. The particulate nature of CB and the presence of CB agglomerates observed in Figure 2 may have contributed to the formation of relatively stable and redundant conductive pathways.

The substantially higher sensitivity of the TBHPS was likely associated with its hybrid conductive network. In addition to CB–CB and CNF–CNF interactions, CB–CNF junctions were distributed throughout the sensing layer as a result of high-shear planetary mixing, as shown in Figure 4. Thus, three types of conductive junctions, namely CB–CB, CNF–CNF, and CB–CNF, can contribute to the piezoresistive response of the TBHPS. Changes in the resistance of these junctions during deformation may result in a more complex and strain-sensitive conductive network.

As previously mentioned, spherical CB particles with an aspect ratio of approximately 1 respond to deformation differently from fibrous CNFs. CB particles can move either toward or away from neighboring particles during deformation, whereas CNFs can undergo additional geometrical changes. As shown in Figure 3 and Figure 4, the CNFs exhibited no dominant orientation throughout the sensing layer. During deformation of the SEBS matrix, individual CNFs may not only move toward or away from neighboring conductive fillers, but may also rotate, slip, align, and reorient owing to their fibrous geometry and the dimensional changes in the matrix. As illustrated in Figure 5a, the sensing mechanism of the TBHPS is therefore likely governed by the synergistic interactions among CB particles and CNFs. These combined deformation mechanisms and changes in CB–CB, CNF–CNF, and CB–CNF conductive junctions may account for the high and stable sensitivity of the TBHPS, consistent with the synergistic effects of hybrid conductive fillers previously reported in the literature [3,52,53,54].

**Figure 7:**
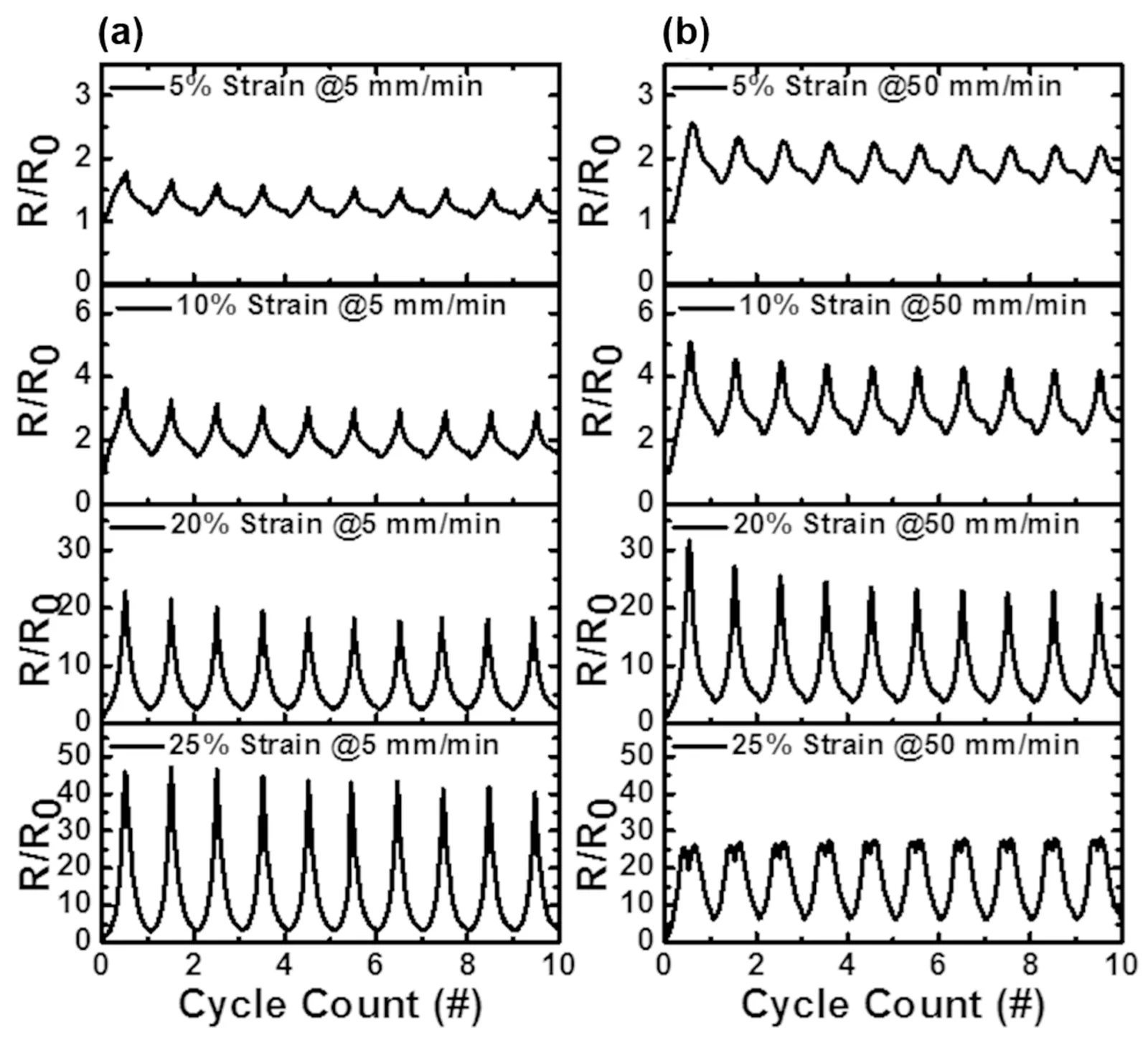
R/R_0_ vs. cycle number for TBHPS at (**a**) 5 mm/min and (**b**) 50 mm/min for 10 cycles at different strain levels (5–25%).

**Figure 8:**
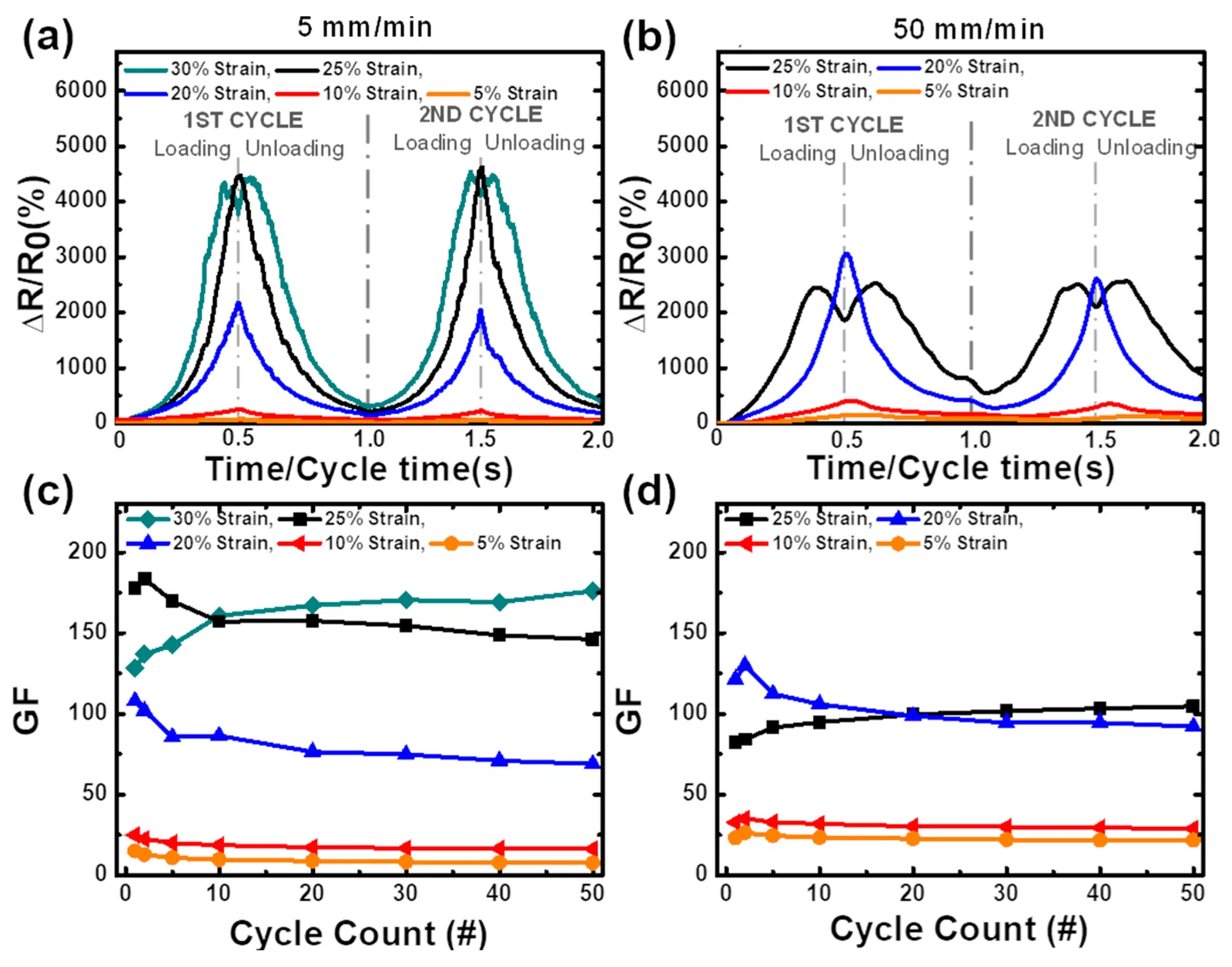
∆R/R_0_ (%) vs. time/cycle time under different strain levels (5–30%) for initial 2 cycles of TBHPS with test speeds of (**a**) 5 mm/min and (**b**) 50 mm/min; GF (**c**) at 5 mm/min and (**d**) 50 mm/min.

TBHPS was tested for 50 cycles at strain levels of 5, 10, 20, 25, and 30% at a crosshead speed of 5 mm/min and at strain levels of 5, 10, 20, and 25% at 50 mm/min. In this way, the effects of both strain magnitude and deformation speed on the sensing performance were investigated. The strain- and speed-dependent relative piezoresistive responses of the hybrid textile sensors are presented in Figure 7 for the first 10 cycles in terms of relative resistance (R/R_0_). As shown in Figure 7, and Figure 8a,b, at 5 mm/min, the resistance increased during tensile loading, and the magnitude of the resistance change increased with increasing strain. The R/R_0_ values were approximately 1.5, 3, 20, and 45 at strain levels of 5, 10, 20, and 25%, respectively. However, at 30% strain, the piezoresistive behavior changed from PP to NP at approximately 26% strain (Figure 8a). When the test speed increased to 50 mm/min, a similar strain-dependent response was observed, with R/R_0_ values of approximately 2.2, 4, and 25 at strain levels of 5, 10, and 20%, respectively. At 25% strain, however, the response changed from PP to NP at approximately 21% strain (Figure 8b). Therefore, the upper strain limits for maintaining a positive piezoresistive response were approximately 25% at 5 mm/min and 20% at 50 mm/min. These results indicate that the effective positive-piezoresistive operating range of the sensor was extended at the lower deformation speed.

**Figure 9:**
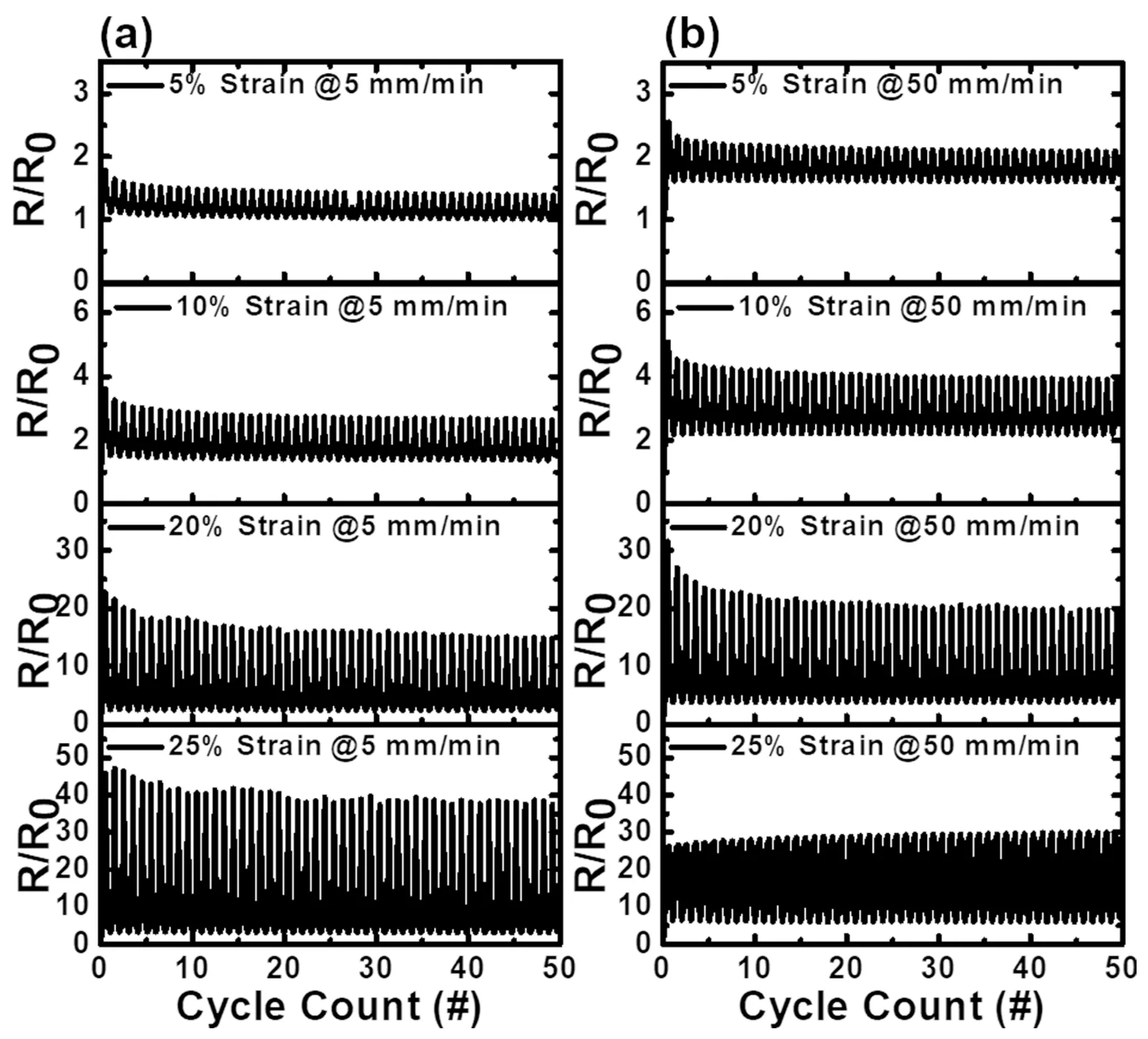
R/R_0_ vs. cycle number for TBHPS at (**a**) 5 mm/min and (**b**) 50 mm/min for 50 cycles at different strain levels (5–25%).

Another important performance parameter is cyclic sensing stability, which reflects the consistency of the sensor response during repeated loading–unloading cycles. The cyclic responses of the TBHPS at 5 and 50 mm/min are presented in Figure 9. The hybrid textile sensor exhibited generally consistent cyclic response profiles at both deformation speeds after the pronounced changes observed during the initial cycles. Sensor recovery can be further evaluated by comparing the resistance responses obtained during loading and unloading, with closer agreement between the two branches indicating lower electrical hysteresis. To quantify this behavior, the electrical hysteresis magnitude was calculated using the area-based method described in Section 2. For the representative 10th loading–unloading cycle, the hysteresis magnitudes at 5 mm/min were 6.9, 3.3, 13.9, and 18.9% at maximum strains of 5, 10, 20, and 25%, respectively. At 50 mm/min, the corresponding values were 19.2, 15.9, 36.5, and 22.1% (Figure S4). Thus, the hysteresis magnitude was consistently higher at 50 mm/min than at 5 mm/min at all investigated strain levels, confirming the poorer electrical recovery observed at the higher deformation speed. The hysteresis response also depended on cycle number, with the first cycle showing markedly higher values under several test conditions. Therefore, hysteresis values for the 1st, 5th, 10th, 15th, and 20th cycles, together with the corresponding initial resistance values (R_0_), are provided in the Supporting Information (Table S1). The improved recovery observed at 5 mm/min may be associated with the longer time available for the viscoelastic relaxation of the SEBS-based sensing layer and rearrangement of the conductive fillers during unloading. In contrast, the shorter loading–unloading period at 50 mm/min may restrict structural recovery of the sensing layer, contributing to greater loading–unloading asymmetry and increased unrecovered strain [51].

The GF values determined from the first loading cycles were approximately 15, 25, 108, and 178 at strain levels of 5, 10, 20, and 25%, respectively, at a crosshead speed of 5 mm/min. At 50 mm/min, the corresponding GF values were approximately 23, 33, and 122 at strain levels of 5, 10, and 20%, respectively. Under both test conditions, GF increased with increasing strain. As shown in Figure 7 and Figure 8, the sensor exhibited a larger relative resistance change at 50 mm/min than at 5 mm/min. On the other hand, the electrical recoverability of the hybrid sensor was lower at 50 mm/min, as indicated by the loading–unloading responses presented in Figure 9. Since R/R_0_ is normalized to its initial resistance, the first value is the unity for all measurements. Therefore, recoverability was evaluated based on the extent to which R/R_0_ returned toward unity after unloading. At 5 mm/min, the post-unloading R/R_0_ values were generally closer to 1 than those obtained at 50 mm/min, indicating improved resistance recovery at the lower deformation speed. This behavior may be associated with the strain-rate-dependent viscoelastic response of the SEBS matrix. At higher deformation rates, reduced molecular-chain mobility can result in a stiffer mechanical response and provide less time for relaxation and structural recovery of both the polymer matrix and the conductive filler network. Consequently, rearrangement of CB and CNFs during unloading may be more limited at 50 mm/min, leading to larger residual resistance changes. In terms of cyclic sensing stability, the GF response did not change markedly after the 10th cycle under the investigated test conditions.

**Figure 10:**
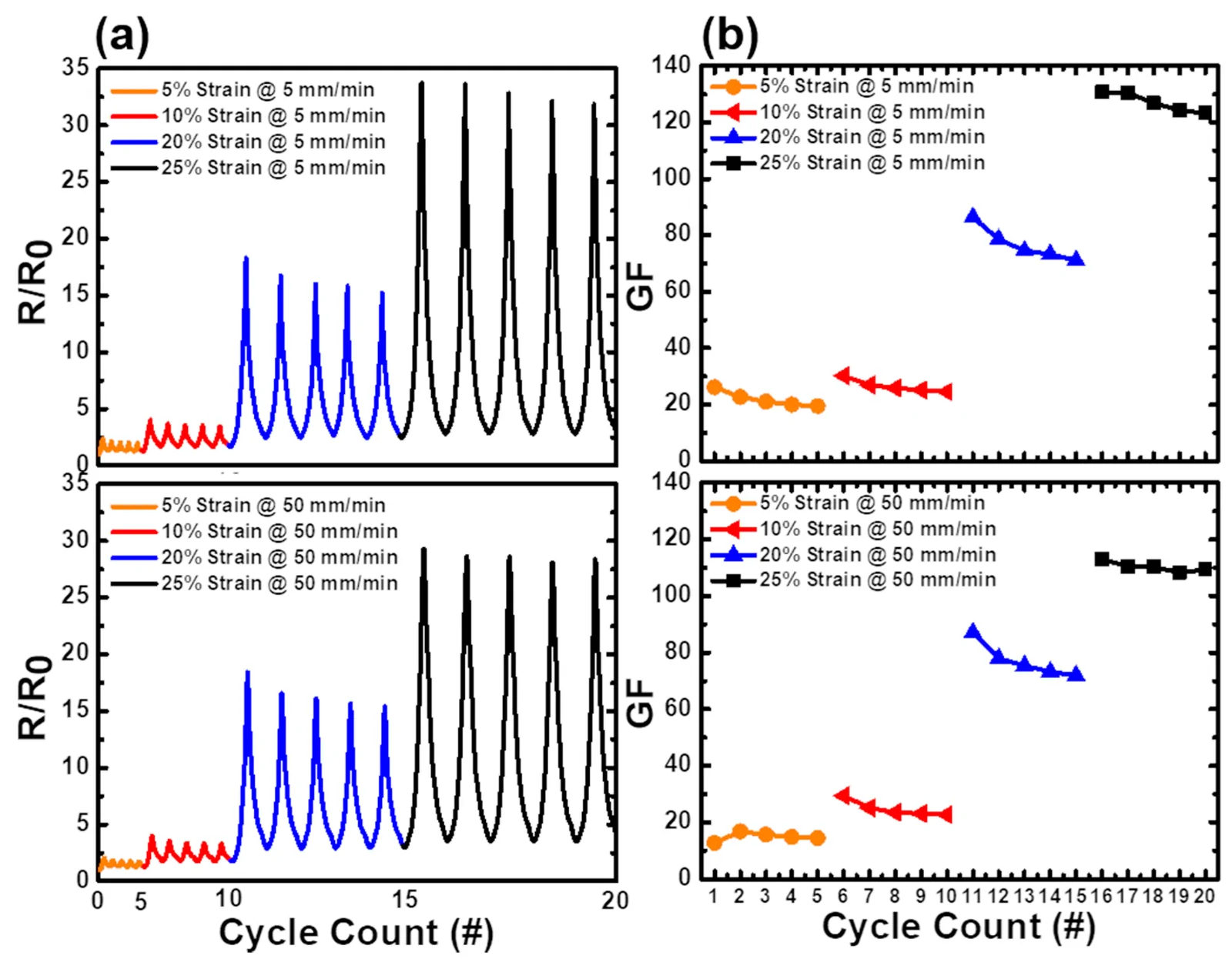
Stepwise piezoresistance test for TBHPS: (**a**) R/R_0_ at 5 mm/min and 50 mm/min and (**b**) GF at 5 mm/min and 50 mm/min.

In order to analyze the behavior of the TBHPS under different strain levels, the sensor was subjected to five repeated stepwise loading–unloading cycles at strain levels of 5, 10, 20, and 25%. Figure 10 presents the R/R_0_ responses as a function of cycle number at test speeds of 5 and 50 mm/min. Regardless of the applied strain level, the TBHPS exhibited a positive piezoresistive response, and the sensitivity increased with increasing strain. Unlike the separate constant-strain tests, no transition from positive-to-negative piezoresistance was observed under the stepwise loading conditions. This difference may be associated with the gradual increase in and release of strain during stepwise cycling, which may provide additional time for viscoelastic relaxation and the reorganization of the conductive network between successive strain levels. As shown in Figure 10b, the GF values obtained over five cycles at 5 mm/min are in the ranges of 19–26, 24–30, 71–86, and 123–130 at strain levels of 5, 10, 20, and 25%, respectively. At 50 mm/min, the corresponding GF ranges were 12–24, 22–29, 71–87, and 109–113. Under both test conditions, GF increased with increasing strain. The GF values obtained at 5 mm/min were slightly higher at 5, 10, and 20% strain and noticeably higher at 25% strain compared with those obtained at 50 mm/min. As discussed previously, this behavior may be associated with the improved recoverability of the hybrid textile sensor at the lower deformation speed. To further assess sensing stability, the % change in GF between the 1st and 5th cycles was calculated. At 5 mm/min, the GF changes were approximately 27, 20, 11.3, and 5.4% at strain levels of 5, 10, 20, and 25%, respectively. At 50 mm/min, the corresponding values were approximately 15, 25, 18, and 3.5%. In general, the magnitude of the GF change decreased at higher strain levels, indicating improved cycle-to-cycle stability. Although both deformation speeds showed a similar overall trend, the 5 mm/min condition exhibited a more systematic decrease in GF variation with increasing strain.

### 3.3. Mechanical Properties

**Figure 11:**
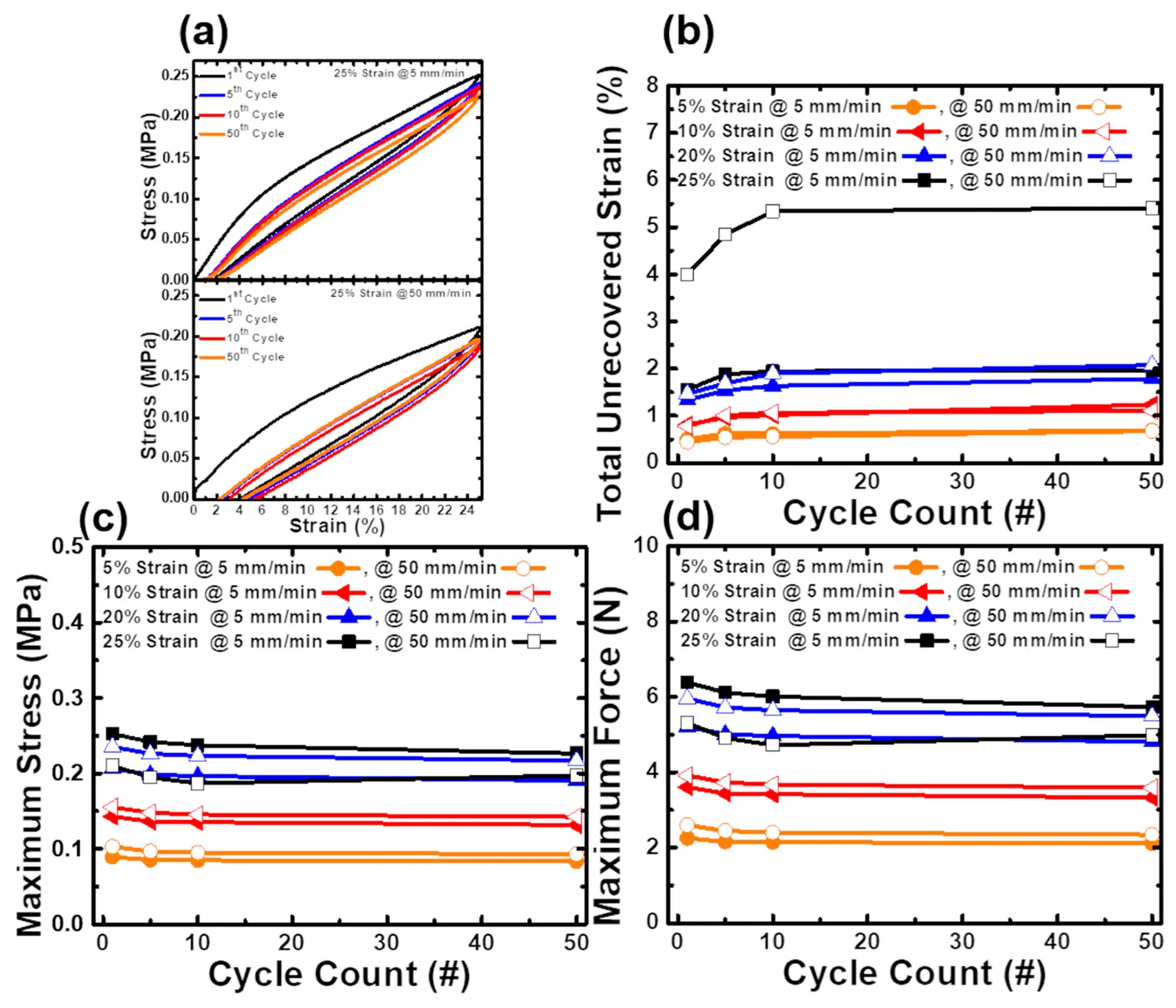
(**a**) Mechanical cycling of TBHPS at 5 mm/min and 50 mm/min, (**b**) unrecovered strain at 5 mm/min and 50 mm/min, (**c**) stress softening at 5 mm/min and 50 mm/min, and (**d**) maximum force at 5 mm/min and 50 mm/min.

For piezoresistive sensors, not only electrical but also mechanical properties are important for understanding the performance limits of the sensing system. From this perspective, the stress–strain responses of the hybrid textile sensor subjected to 25% cyclic strain at test speeds of 5 and 50 mm/min are presented for the 1st, 5th, 10th, and 50th cycles in Figure 11a. The stress–strain curves provide information on unrecovered strain, stress softening, and maximum force. Figure 11b–d were constructed using data extracted from the stress–strain curves obtained over 50 loading–unloading cycles. The hybrid textile sensor exhibited decreases in stress and maximum force under all test conditions (Figure 11c,d). This behavior is characteristic of the Mullins effect, which is commonly observed in elastomeric composites subjected to repeated cyclic loading and unloading at a constant strain level. During cyclic deformation, various reversible and irreversible interactions may occur among the polymer chains, conductive fillers, and the polymer–filler interfaces. Polymer chains may undergo sliding, rearrangement, and changes in orientation, while deformation of the polymer matrix can alter the spatial organization of the conductive fillers. Accordingly, the fillers may undergo rotation, slippage, translation, uncurling, alignment, reorientation, and separation during deformation. The filler–matrix interface also plays an important role in the mechanical response. Because stress transfer between the fillers and the polymer matrix occurs through the interface, cyclic deformation may induce complex interfacial rearrangements and, in some cases, partial disruption of polymer chains or polymer–filler interactions near the interface. These structural changes contribute to stress softening during repeated cycling. At the end of unloading, residual deformation may remain even when the applied stress returns to zero, resulting in unrecovered strain and preventing the complete recovery of the original sample dimensions. The magnitude of unrecovered strain depends on factors such as the mechanical properties of the composite, filler content, applied strain level, and deformation speed [3,51,55,56,57,58]. In this study, the unrecovered strain is expected to originate from the combined mechanical response of both the conductive sensing layer and the underlying textile substrate.

As shown in Figure 11, the total unrecovered strain increased with increasing strain level, reflecting the greater extent of deformation. Most of the permanent deformation developed during the first unloading cycle, whereas the rate of additional strain accumulation and stress softening decreased during subsequent cycles. At strain levels between 5 and 20%, the effect of deformation speed on unrecovered strain was relatively limited. However, at 25% strain, testing at 50 mm/min resulted in a substantially higher unrecovered strain than testing at 5 mm/min (Figure 11b). This behavior may be associated with the reduced time available for viscoelastic recovery at the higher deformation speed. After 50 cycles at 25% strain, the total unrecovered strain values of the neat fabric were 1.45 and 1.46% at 5 and 50 mm/min, respectively. In contrast, the corresponding values for the hybrid textile sensor were 1.97 and 5.78%. These results indicate that incorporation of the conductive sensing layer increased the unrecovered strain, particularly at the higher deformation speed. This increase may be attributed to the higher stiffness of the TBHPS and its lower recoverability compared with the neat elastic knitted fabric.

**Figure 12:**
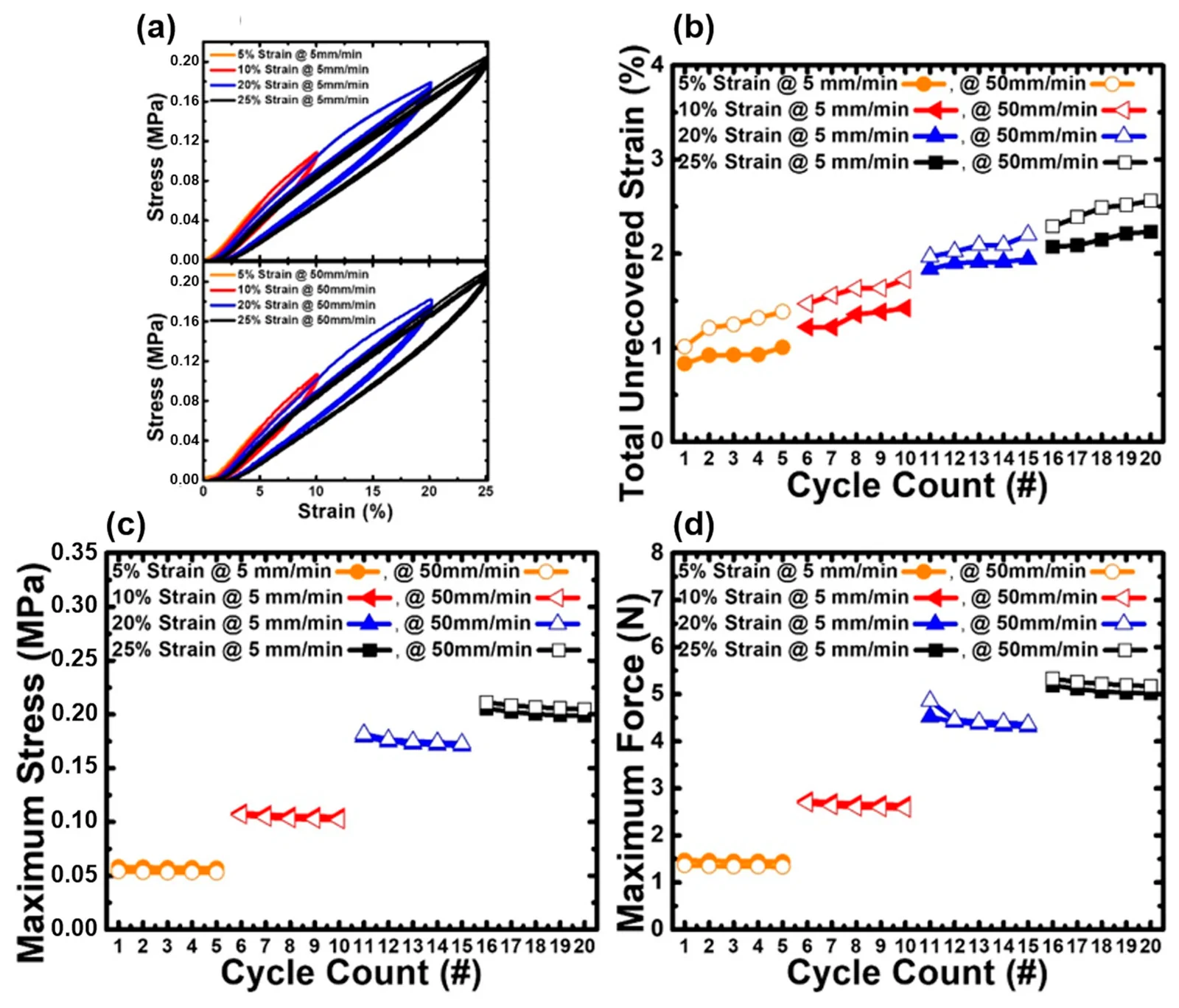
Stepwise test of TBHPS: (**a**) mechanical cycling at 5 mm/min and 50 mm/min, (**b**) unrecovered strain at 5 mm/min and 50 mm/min, (**c**) stress softening at 5 mm/min and 50 mm/min, and (**d**) maximum force at 5 mm/min and 50 mm/min.

The mechanical cycling data corresponding to the stepwise piezoresistive tests presented in Figure 10 are shown in Figure 12. As shown in Figure 12, stress softening and a reduction in maximum force were observed as functions of both applied strain and cycle number. Consistent with these observations, the unrecovered strain increased with increasing cycle number and applied strain level, reflecting the cyclic stress-softening behavior associated with the Mullins effect discussed in the previous section. The accumulation of unrecovered strain was more pronounced at 50 mm/min, particularly at higher strain levels. This behavior may be attributed to the shorter time available for viscoelastic recovery of the SEBS-based sensing layer and the elastic textile substrate at the higher deformation speed. Since the test speed at 50 mm/min is ten times that at 5 mm/min, the time available for loading and unloading to a given strain level is approximately one-tenth under identical test conditions. Consequently, the polymer chains and conductive network have less time for relaxation and structural rearrangement, which may contribute to the higher unrecovered strain observed at 50 mm/min.

### 3.4. Human-Motion Monitoring

**Figure 13:**
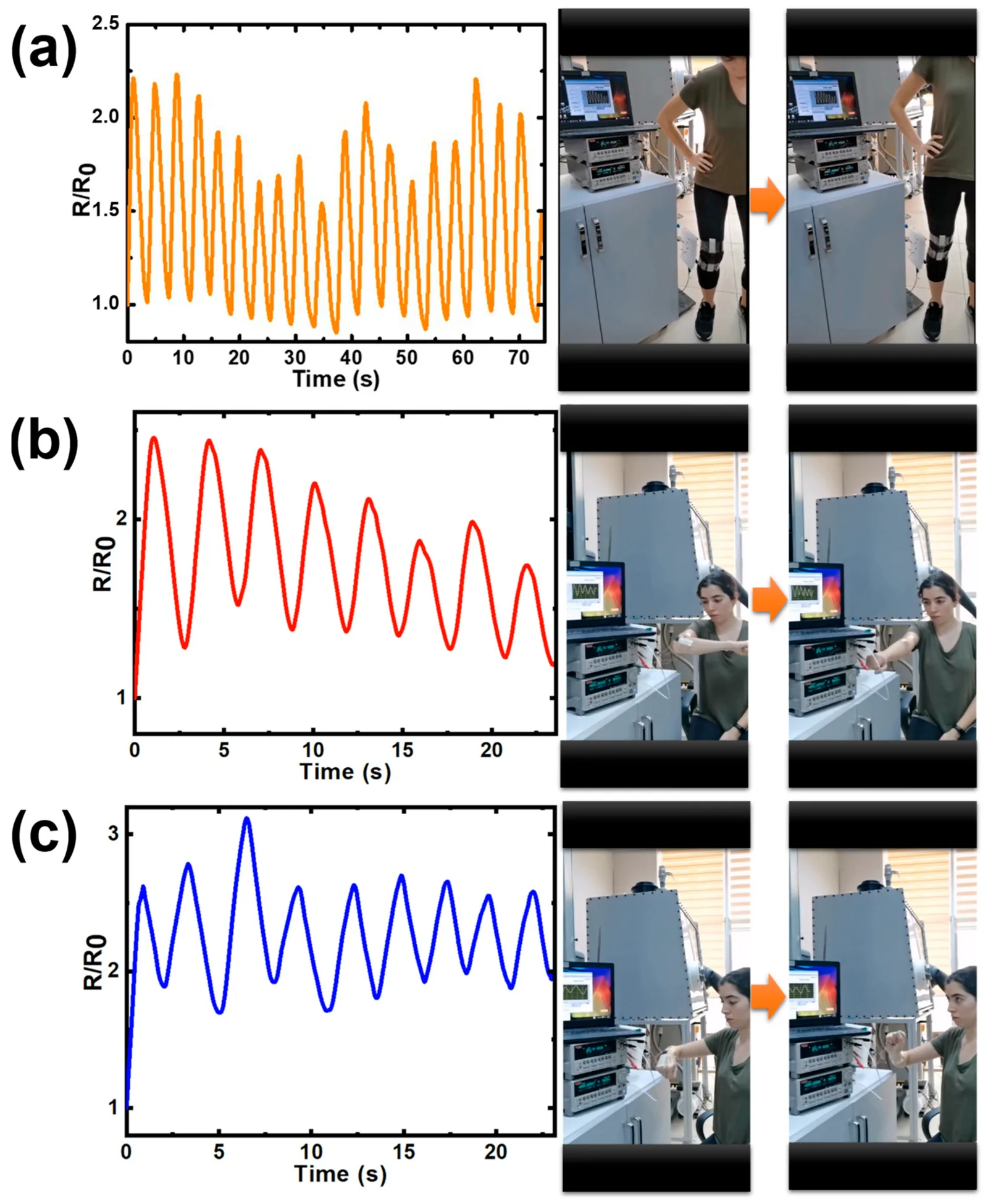
TBHPS capable of monitoring the movements of a knee during cyclic (**a**) squat, (**b**) elbow, and (**c**) wrist exercises (see Supplementary Videos).

Human-motion tracking is an important technology for the development of advanced systems for personalized health monitoring and human–machine interaction. As demonstrated in the previous sections, the TBHPS developed in this study combines stretchability, flexibility, bendability, high sensitivity, and good resilience. To evaluate its capability for monitoring human motion, the sensor was attached to different joints of a volunteer, including the wrist, elbow, and knee, and various body movements were performed, as shown in Figure 13. The sensor produced reproducible responses during repeated cyclic movements at all joint locations investigated. These results demonstrate the potential of the developed TBHPS for human-motion monitoring applications.

## 4. Discussion and Conclusions

In this study, a highly elastic knitted fabric with a flexible hybrid sensing layer was used to develop textile-based hybrid piezoresistive sensing technology for human-motion monitoring. The main findings can be summarized as follows:

- Three different textile sensors were prepared by blade coating of CB/SEBS, CNF/SEBS, and hybrid CB+CNF/SEBS conductive pastes.
- Cross-sectional FESEM observations showed continuous coverage of the textile surface by the SEBS-based sensing layers, good interfacial contact with the fabric, and satisfactory dispersion of the conductive fillers. In the hybrid sensing layer, CB particles were observed between CNFs, resulting in CB–CB, CNF–CNF, and CB–CNF junctions within the conductive network.
- Regardless of the filler type, strain-induced reproducible and reversible positive piezoresistive responses were observed for all three sensors. At 20% strain and a deformation speed of 5 mm/min, the approximate GF values were 0.2, 45, and 108 for the CB/SEBS, CNF/SEBS, and hybrid CB+CNF/SEBS sensors, respectively, demonstrating the substantially higher strain sensitivity of the hybrid formulation.
- The piezoresistive behavior of the hybrid sensor depended on both strain level and deformation speed. Positive piezoresistive sensing was maintained up to approximately 25% strain at 5 mm/min and 20% strain at 50 mm/min, whereas a transition toward negative piezoresistance occurred at higher strain levels.
- The enhanced sensitivity of the hybrid sensor is consistent with the formation of a heterogeneous conductive network containing CB–CB, CNF–CNF, and CB–CNF junctions and the complementary deformation behavior of particulate CB and fibrous CNFs. However, this contribution should be considered a mechanistic interpretation rather than direct proof of an isolated CB–CNF synergistic effect.
- The hybrid sensor exhibited stable cyclic resistance responses under repeated loading and unloading. Lower deformation speed resulted in improved recoverability, whereas the larger response observed at a higher deformation speed was accompanied by increased unrecovered strain, indicating rate-dependent electromechanical behavior.
- The TBHPS developed in this study provides a promising platform for body-compatible wearable sensing by enabling the monitoring of wrist, elbow, and knee movements. The sensor combines flexibility and conformability while largely preserving the inherent characteristics of the textile substrate. In addition, its electrical response can be tailored through conductive-filler selection, and the blade-coating process offers a relatively simple fabrication route with potential for batch processing and integration with other functional coatings.

## Data Availability

The data presented in this study are available on request from the corresponding authors. The data are not publicly available because no dedicated public repository was used for this study. Supplementary Materials, including the videos related to human-motion monitoring, are available with the published article.

## Supplementary Materials

The following supporting information can be downloaded at https://www.mdpi.com/article/10.3390/polym18182205/s1, Supporting Information: Additional experimental data, characterization results, and supporting information related to the performance of the developed textile-based piezoresistive sensors; Figure S1. Cross-sectional FESEM images of the CB-based textile sensor. Circles: CB; Figure S2. Cross-sectional FESEM images of the CNF-based textile sensor. Arrows: CNF; Figure S3. Cross-sectional FESEM images of TBHPS. Arrows: CNF and dotted-line circles: CB; Figure S4. Calculated electrical hysteresis magnitude (DH) of the TBHPS at 5, 10, 20, and 25% strain for the 10th loading–unloading cycle at deformation speeds of 5 and 50 mm/min; Table S1. Initial resistance and electrical hysteresis magnitude (DH%) of the hybrid CB+CNF/SEBS textile sensor at selected loading–unloading cycles under different strain amplitudes and deformation rates; Video S1: Hybrid fabric sensor for human motion monitoring during elbow movement; Video S2: Hybrid fabric sensor for human motion monitoring during squatting; Video S3: Hybrid fabric sensor for human motion monitoring during wrist movement.

## Abbreviations

The following abbreviations are used in this manuscript:

CB	Carbon black
CNC	Cellulose nanocrystal
CNF	Carbon nanofiber
CNT	Carbon nanotube
Co	Cotton
FESEM	Field-emission scanning electron microscopy
GF	Gauge factor
GNPs	Graphene nanoplatelets
ICPs	Inherently conductive polymers
MWCNT	Multi-wall carbon nanotube
NP	Negative piezoresistance
PANI	Polyaniline
PDA	Polydopamine
PDMS	Polydimethylsiloxane
PEDOT	Poly(3,4-ethylenedioxythiophene) sulfonate
PET	Polyethylene terephthalate
PP	Positive piezoresistance
PPy	Polypyrrole
rGO	Reduced graphene oxide
SEBS	Poly[styrene-b-(ethylene-co-butylene)-b-styrene]
SWCNT	Single-wall carbon nanotube
TBHPS	Textile-based hybrid piezoresistive sensor
TBPS	Textile-based piezoresistive sensor
TPU	Thermoplastic polyurethane
