# Supplementary material for: Flexible Textile-Based Hybrid Piezoresistive Sensors for Human-Motion Monitoring
Source: Polymers (Basel). 2026 Sep 10;18(18):2205. doi: 10.3390/polym18182205 (PMC13611405; doi:10.3390/polym18182205)
Supplement: Supplementary file 1 [file polymers-18-02205-s001.zip › supporting information.pdf]

SUPPORTING INFORMATION

Flexible Textile-Based Hybrid Piezoresistive Sensors for Human Motion Monitoring

Hatice Aylin Karahan Toprakci[1,2,*], Mukaddes Sevval Cetin[2] and Ozan Toprakci[1,2]

[1] Yalova University Department of Polymer Materials Engineering, Yalova, Turkey
[2] Yalova University, Institute of Graduate Studies, Yalova, Turkey
* Correspondence: aylin.toprakci@yalova.edu.tr; Tel.: +90 551 553 3916

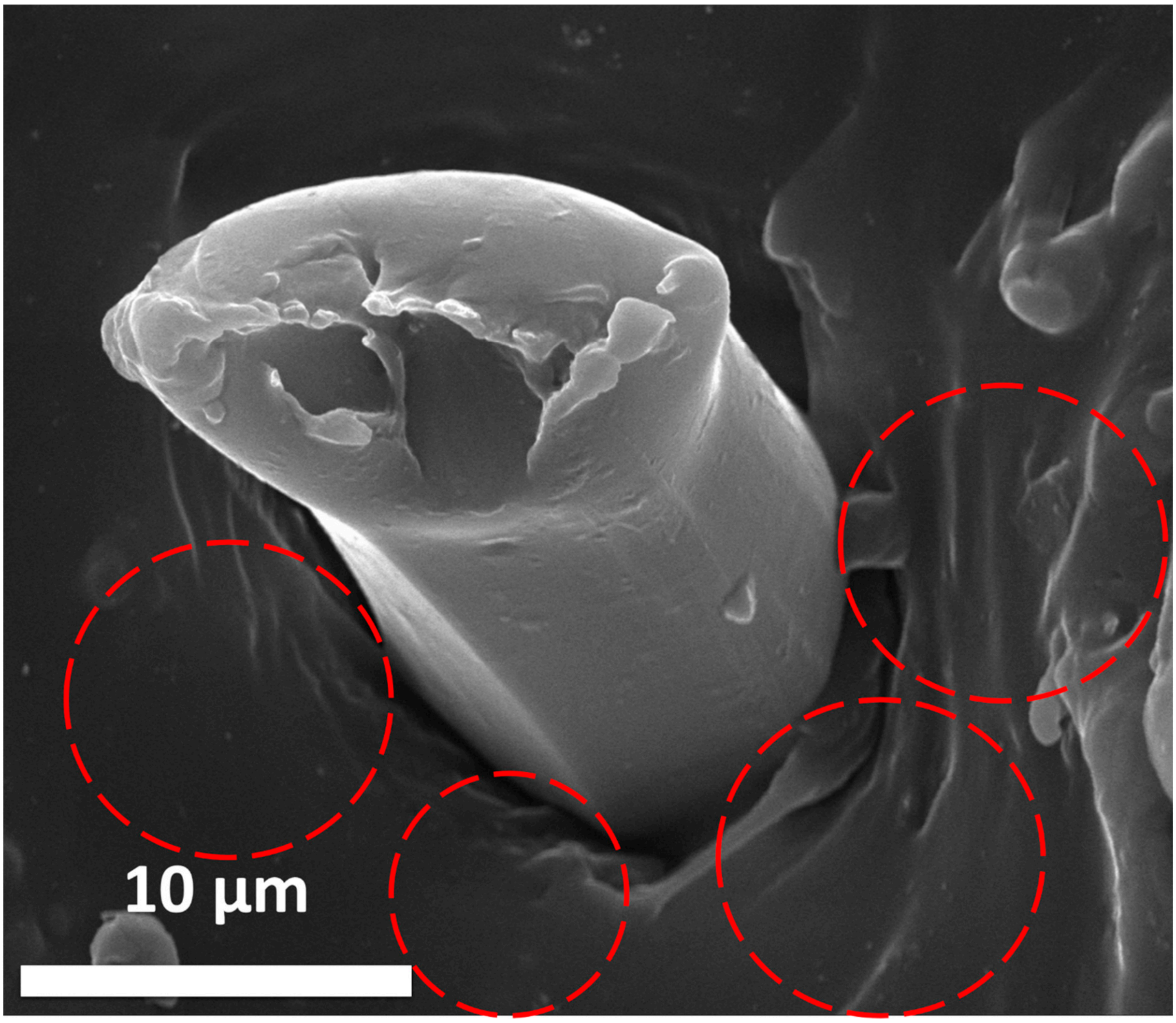

Figure S1. Cross-sectional FESEM images of the CB-based textile sensor. Circles: CB

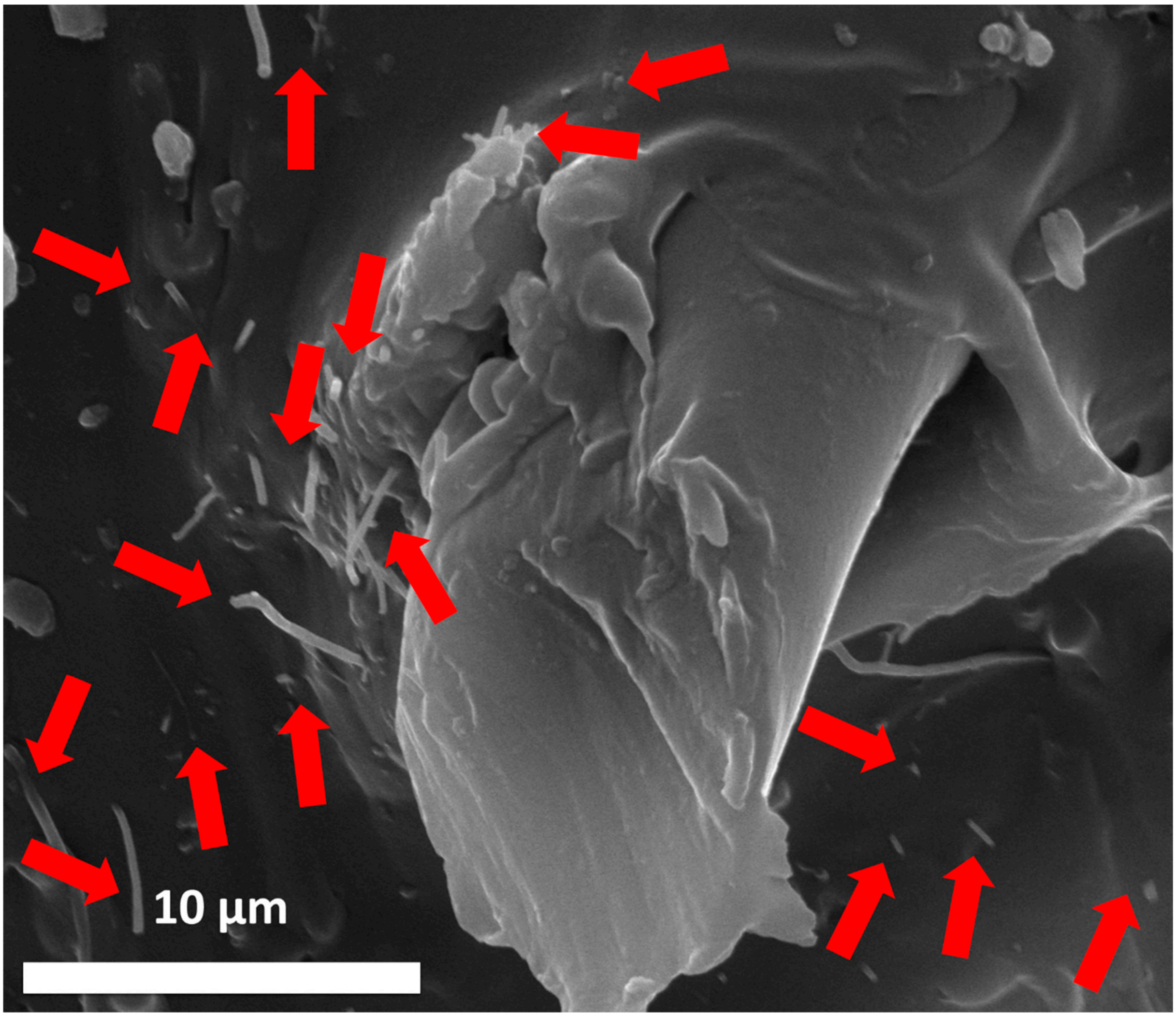

Figure S2. Cross-sectional FESEM images of the CNF-based textile sensor. Arrows: CNF

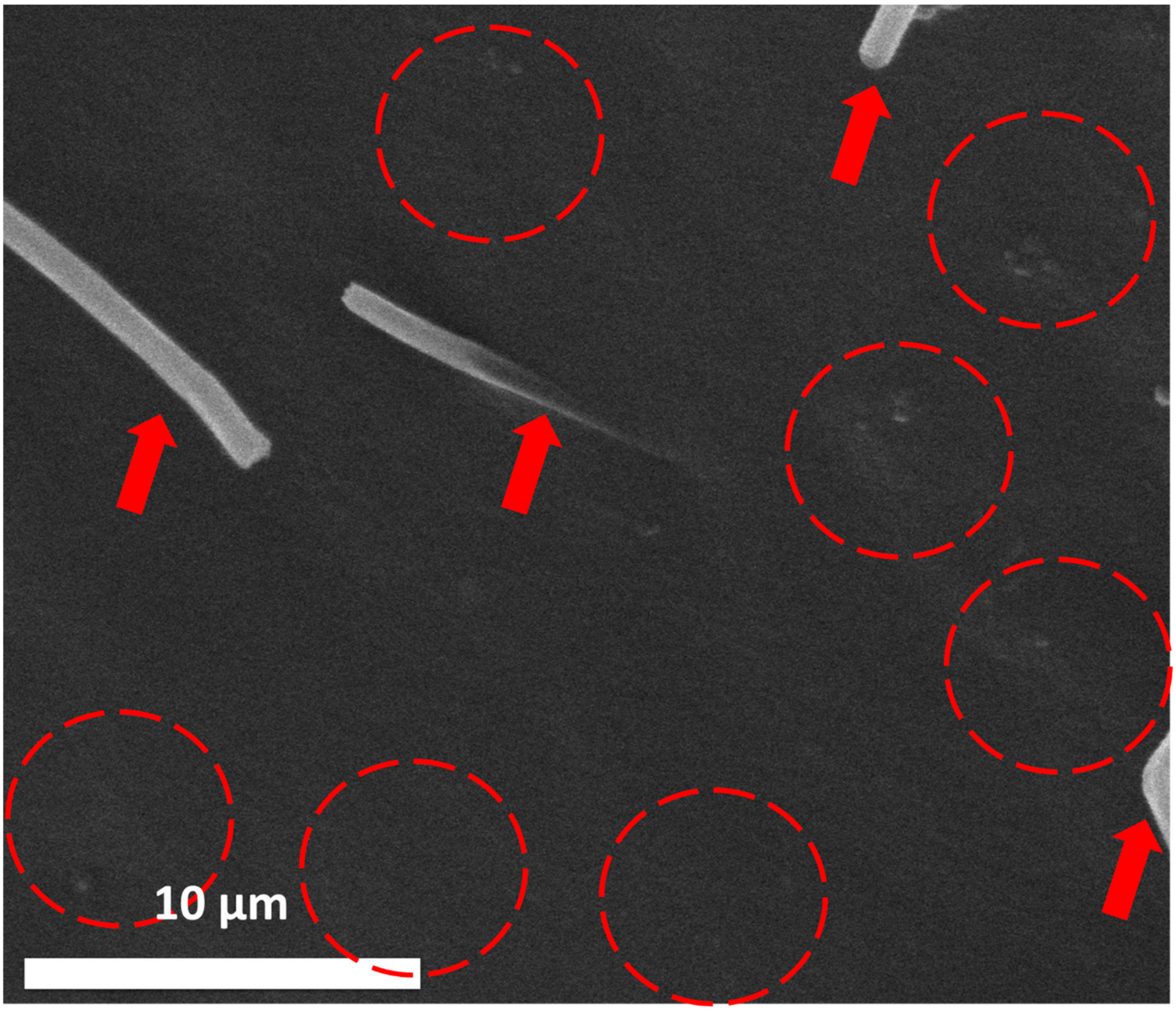

Figure S3. Cross-sectional FESEM images of TBHPS. Arrows: CNF and dotted-line circles: CB

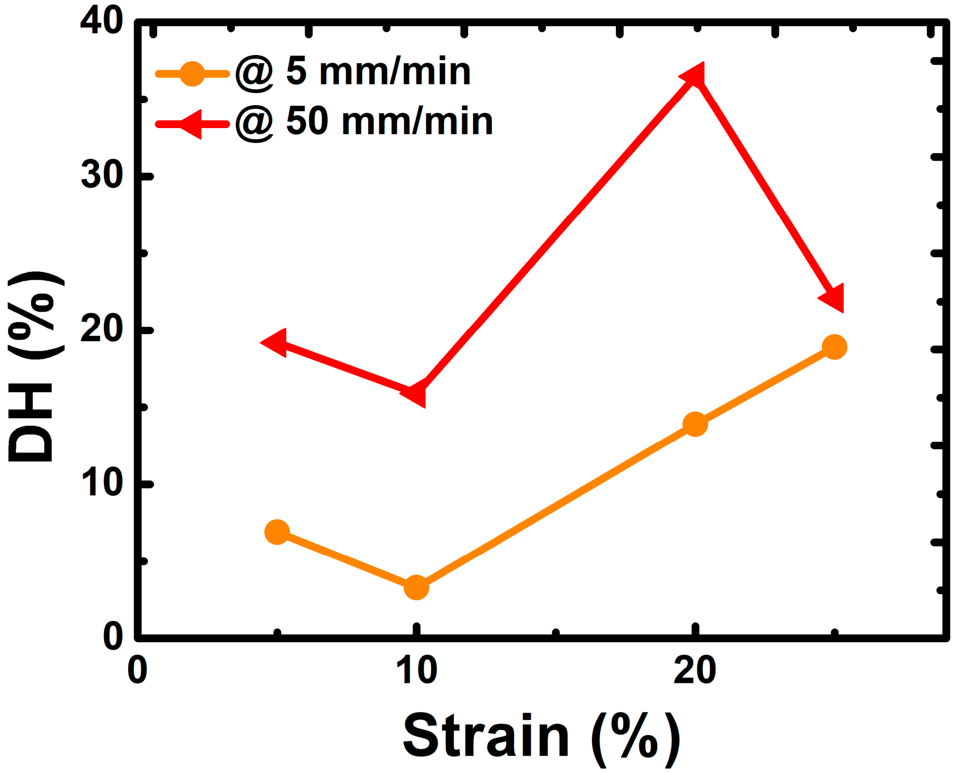

Figure S4. Calculated electrical hysteresis magnitude (DH) of the TBHPS at 5, 10, 20, and 25% strain for the 10th loading–unloading cycle at deformation speeds of 5 and 50 mm/min.

To examine the influence of cyclic conditioning on the electrical hysteresis response, the hysteresis magnitude was calculated for the 1st, 5th, 10th, 15th, and 20th loading–unloading cycles under each strain amplitude and deformation rate. The corresponding initial resistance values (R_0) used for normalization are also provided in Table S1.

Table S1. Initial resistance and electrical hysteresis magnitude (DH%) of the hybrid CB+CNF/SEBS textile sensor at selected loading–unloading cycles under different strain amplitudes and deformation rates.

Maximum strain (%)	Speed (mm/min)	R_0 (Ω)	DH (%)				
			1st cycle	5th cycle	10th cycle	15th cycle	20th cycle
5	5	87,725	9.4	5.6	6.9	7.4	9.5
10	5	88,640	2.0	2.9	3.3	3.7	3.5
20	5	65,791	44.7	11.7	13.9	8.3	10.4
25	5	85,726	35.5	17.5	18.9	16.7	14.0
5	50	65,972	86.4	21.7	19.2	18.6	18.2
10	50	77,366	49.5	16.4	15.9	15.4	14.2
20	50	76,703	60.2	39.2	36.5	36.6	34.7
25	50	94,224	40.0	21.9	22.1	22.1	20.6

Note: R_0 represents the initial electrical resistance recorded at the beginning of each test before cyclic deformation. The normalized resistance change was calculated as $\Delta R/R_0 = (R - R_0)/R_0$. The hysteresis magnitude was calculated as

$$DH = | A_{\mathrm{loading}} - A_{\mathrm{unloading}} | / A_{\mathrm{loading}} \times 100,$$

where A_{loading} and $A_{\mathrm{unloading}}$ are the integrated areas under the loading and unloading branches of the normalized resistance–strain response, respectively.

As shown in Table S1, the first loading–unloading cycle exhibited pronounced hysteresis under several conditions, particularly at 20 and 25% strain at 5 mm/min and across the investigated strain range at 50 mm/min. After the initial cycle, the hysteresis values were generally reduced and remained within a narrower range over the selected subsequent cycles. Moreover, the hysteresis values at 50 mm/min were consistently higher than those at 5 mm/min for the representative 10th cycle, indicating a clear rate dependence of the electrical loading–unloading response.
